# A comprehensive nationwide registry study of noncommunicable disease comorbidities and death in cancer patients in Norway—the NCDNOR project

**DOI:** 10.1038/s41598-026-41831-6

**Published:** 2026-02-28

**Authors:** Simon Lergenmuller, Trude Eid Robsahm, Yngvar Nilssen, Knut Eirik Dalene, Wenche Nystad, Haakon E. Meyer, Hein Stigum, Vidar Hjellvik, Lars J. Kjerpeseth, Inger Ariansen, Inger Kristin Larsen

**Affiliations:** 1 Department of Registration, Cancer Registry of Norway, Norwegian Institute of Public Health, P.O Box 5313, Majorstuen, 0304 Oslo, Norway; 2 Present Address: Department of Method Development and Analytics, Norwegian Institute of Public Health, P.O Box 222, Skøyen, 0213 Oslo, Norway; 3 Department of Research, Cancer Registry of Norway, Norwegian Institute of Public Health, P.O Box 5313, Majorstuen, 0304 Oslo, Norway; 4 Department of Chronic Diseases, Norwegian Institute of Public Health, P.O Box 222, Skøyen, 0213 Oslo, Norway; 5 Department of Physical Health and Ageing, Norwegian Institute of Public Health, P.O Box 222, Skøyen, 0213 Oslo, Norway; 6 Department of Community Medicine and Global Health, University of Oslo, P.O. Box 1130, Blindern, 0318 Oslo, Norway

**Keywords:** Cancer comorbidities, Noncommunicable diseases, Multi-state models, Cohort study, National registries, Comorbidity patterns, Cancer, Diseases, Health care, Medical research, Oncology, Risk factors

## Abstract

Noncommunicable disease (NCD) comorbidities are common in cancer patients due to shared risk factors, but few studies have mapped their occurrence across cancer types using detailed national data. In this nationwide cohort study, we used data from Norwegian mandatory health registries to comprehensively describe NCD comorbidities at and after cancer diagnosis of 19 cancers. We included 269,956 adults registered with a first cancer in the Cancer Registry of Norway 2009–2019. Comorbidities included second cancers, cardiovascular diseases (CVDs), chronic obstructive pulmonary disease, diabetes and mental health disorders (MDs). We used intersection diagrams to examine NCD comorbidity patterns at first cancer diagnosis, and multi-state models to estimate five-year post-cancer probabilities of NCD comorbidities and death. We also provide a publicly available online application [https://ncdapp.onrender.com/] with additional results and details. Comorbidity prevalence at cancer diagnosis ranged from 35% (skin cancer) to 83% (lung cancer). CVD was most prevalent, usually co-occurring with MDs or diabetes. Lowest prevalences were found for younger patients and women. Conditioning on surviving five years, comorbidity probability exceeded 50% in all sites. Our findings show substantial comorbidity burden in cancer patients, varying by cancer site, age, and sex. Knowledge of these patterns could improve NCD prevention, treatment and surveillance.

**Supplementary Information:**

The online version contains supplementary material available at 10.1038/s41598-026-41831-6.

## Introduction

Although cancer remains a leading cause of death globally, advancements in detection, diagnostic methods, and treatment have significantly increased survival rates^1,2^. Age is a strong independent risk factor for cancer and other noncommunicable diseases (NCDs) such as cardiovascular diseases (CVDs), diabetes, and chronic respiratory diseases^3–7^. These disease groups share several risk factors, including tobacco use, physical inactivity, alcohol consumption, and unhealthy diets, and together account for 75% of NCD deaths worldwide^8,9^. Mental health disorders (MD) are also common in cancer patients and are associated with poorer quality of life and survival^10–14^. Because of these shared risk factors and recent advancements in cancer treatment, cancer patients are increasingly susceptible to living with chronic NCD comorbidities, which in turn contributes to poorer survival^15–18^, presenting substantial challenges to individuals and healthcare systems^4,16,19^.

Few studies give a comprehensive mapping of NCD comorbidity patterns in cancer patients. A recent systematic review reported high variability in comorbidity prevalence at cancer diagnosis^18^, with few studies examining more than one cancer site, or reporting comorbidity-specific estimates. One study considered multiple cancers and comorbidities^20^, but relied on information from a single data source, possibly underestimating prevalence^21–23^. Studies combining multiple data sources are scarce. Cancer patients may also experience MD^10–12^, yet population-based longitudinal studies of multimorbidity that include MD are limited^24^, and studies mapping MD alongside major chronic NCD comorbidities across multiple cancer sites, using linked routine health data, are scarce.

Similar limitations are observed in studies investigating comorbidities post cancer diagnosis^25–27^. Moreover, these studies typically report average measures of association over follow-up (e.g. hazard ratios), which do not provide information on the probabilities of specific comorbidity patterns at specific time since diagnosis. In addition to the competing risk of death^27–30^, accounting for the transitions that patients can experience between different NCD comorbidity patterns over time would provide a clearer picture of the burden of these diseases.

Using data from primary and secondary healthcare from Norwegian nationwide mandatory health registries, we aim to investigate the patterns of NCD comorbidities (second cancer, CVDs, diabetes, chronic obstructive pulmonary disease [COPD], and MDs) in 19 cancer sites by (i) describing NCD comorbidity patterns at first cancer diagnosis and (ii) estimating the probabilities of NCD comorbidity patterns or death at 1–5- and 10-years post-cancer diagnosis. To facilitate easy access to the results, we developed a publicly hosted online application [https://ncdapp.onrender.com/] for clinicians and researchers, which also provides additional details.

## Methods

### Data sources

This study is part of a large project: “A life-course approach to prevent noncommunicable diseases in an ageing population—NCDNOR”, that includes all individuals residing in Norway between 1960 and 2020 (N ~ 10 m)^31^. Cancer patients were identified for the period 1953–2019 using the Cancer Registry of Norway (CRN) according to the International Classification of Diseases 10^th^ revision (ICD-10) (98.7% estimated completeness)^32^. Data on NCD comorbidities (CVD [chronic, excluding hypertension], MD [depression, anxiety], diabetes [type 1 and 2], or COPD) were obtained from nationwide mandatory health registries: The Norwegian Patient Registry (NPR), The Norwegian database for Control and Payment of Health Reimbursement and the Norwegian Prescription Database^31^. We linked the information from the different registries using the unique personal identification number assigned to all Norwegian residents. This allowed follow-up of NCD comorbidity incidence and vital status (alive, emigrated, or dead) by linkage to the Cause of Death registry and Statistics Norway until December 31, 2019. Further details including the exact mapping of each NCD to specific diagnosis or prescription drugs codes can be found in Supplementary Table S1, and details on the data sources included in NCDNOR are described elsewhere^31,33^.

### Study sample

**Fig. 1:**
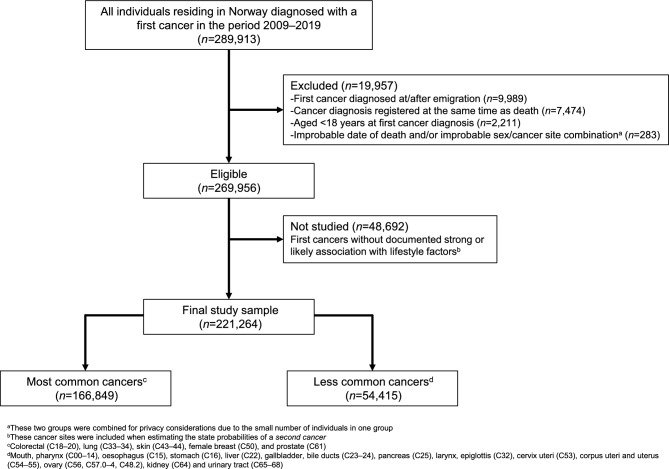
Flowchart illustrating the selection of individuals into the study sample.

We included all individuals diagnosed with a first cancer in the period from January 1, 2009, to December 31, 2019 (*n* = 289,913, Fig. 1), with no previous cancer registered in the CRN in the period 1953–2008. We excluded individuals who emigrated before first cancer diagnosis (*n* = 9,989), individuals with the diagnosis registered at the same time as death (*n* = 7,474), individuals aged < 18 years at the time of first cancer diagnosis (*n* = 2,211), and individuals with improbable date of death and/or improbable sex/cancer site combination (*n* = 283), resulting in 269,956 individuals. We further restricted our sample to individuals whose first cancer diagnosis was at a site with a documented strong or likely association with lifestyle factors (48,692 individuals not studied), resulting in 221,264 individuals aged ≥ 18 years at first cancer diagnosis.

### Statistical analysis

We examined NCD comorbidity patterns at the time of first cancer diagnosis separately for the most common cancers (colorectal [C18–20], lung [C33–34], skin [C43–44], female breast [C50], and prostate [C61]) using intersection diagrams, counting all possible combinations of CVD, MD, diabetes, and COPD registered at or before the first cancer diagnosis. Furthermore, we used multi-state models to estimate stratum-specific (unadjusted beyond stratification) probabilities^34^ of being alive with no, one, two, or three or more comorbidities, or dead, at 1 to 5 years and 10 years post cancer diagnosis (Supplementary Methods A). We also present the probability of being alive with each of the NCD comorbidities (including second cancer) separately. We used the exact timing of registry-defined onsets and follow individuals longitudinally, contributing person-time in each state until a new comorbidity, death, or censoring occurs. Comorbidity patterns were modelled as progressive: once present, they were retained, but patients could transition to states with additional comorbidities. Finally, because the probability of being alive depends on the mortality of the cancer site, we report the corresponding probabilities conditioning on being alive at each time point. Follow-up started from age at first cancer diagnosis, to the date of emigration, death, or end of follow-up (December 31, 2019), whichever occurred first. We used nonparametric bootstrap to compute 95% confidence intervals.

All analyses were stratified by age at diagnosis (18–69 and ≥ 70 years) and sex. We used 70 years as the cut-off for age, as cancer is the first or second leading cause of premature death (deaths occurring < 70 years) in Norway and nearly all other countries with high human development index^35^.

We also report site-specific results for colon (C18), rectal and rectosigmoid (C19–20), melanoma (C43) and nonmelanoma (C44) skin cancers, and for less common cancers, limited to those with documented strong or likely association with lifestyle factors^36,37^: mouth, pharynx (C00–14), oesophagus (C15), stomach (C16), liver (C22), gallbladder, bile ducts (C23–24), pancreas (C25), larynx, epiglottis (C32), cervix uteri (C53), corpus uteri and uterus (C54–55), ovary (C56, C57.0–4, C48.2), kidney (C64) and urinary tract (C65–68).

We also conducted all analyses on a wider sample (*n* = 246,305) where we also included individuals with any prevalent cancers diagnosed on a different site (including those diagnosed in the period 1953–2008). Here, we also allowed individuals with multiple cancer diagnoses to be included in each site-specific analysis (for the cancers diagnosed in the period 2009–2019).

We describe relevant sensitivity analyses in details in Supplementary Methods B. Briefly, sensitivity analyses evaluated robustness to multi-state model specification, age stratification, historical registry availability at baseline, and the choice of assigning comorbidity onset to the first versus confirming registration. All analyses were conducted in R, version 4.2.2 (R Foundation), using the survival- and mstate libraries^38^.

### Ethics declarations

We have thoroughly assessed the ethical and data protection aspects of the NCDNOR project: The Regional Committees for Medical and Health Research Ethics South-East (REK) has approved the project (nr 28561/2019/1203), and a Data Protection Impact Assessment was completed at the Norwegian Institute of Public Health. Data access and linkage approvals were obtained from the relevant data controllers for the registries used in this study. The legal basis for processing of personal data is Article 6 (1) (e) of the GDPR and the exemptions pursuant to Article 9 (2) (j) of the GDPR, with a supplementary legal basis in Sects.  8 and 9 of the Personal Data Act. In accordance with these approvals, and the cited legal bases, individual informed consent for this study using data from mandatory national health registries was not required. All methods were performed in accordance with relevant guidelines and regulations including the Declaration of Helsinki.

## Results

Of the 221,264 individuals, 166,849 were diagnosed with a first cancer of colorectal, lung, skin, female breast or prostate, and 54,415 were diagnosed with a less common cancer (Fig. 1). Median (interquartile range) age at first cancer diagnosis for all sites considered was 68.7 (17.1) years, and for the five most common cancers the median ranged from 61.4 years (female breast) to 71.5 years (colorectal) (Table 1).

**Table 1:** Median age at diagnosis and number of cases per cancer type for women, men and overall.

Cancer type (ICD-10 codes)	Women		Men		Overall	
	No	Median age at diagnosis (IQR)	No	Median age at diagnosis (IQR)	No	Median age at diagnosis (IQR)
**All sites**	103,702	67.4 (21.1)	117,562	69.4 (14.4)	221,264	68.7 (17.1)
**Most common cancers**	75,490	67.3 (21.5)	91,359	69.6 (13.9)	166,849	68.8 (16.8)
**Colorectal (C18**–**20)**	17,307	73.0 (18.0)	17,870	70.2 (16.0)	35,177	71.5 (17.1)
Colon (C18)	12,526	74.1 (17.0)	11,136	71.4 (15.9)	23,662	72.7 (16.6)
Rectum, rectosigmoid (C19–20)	4,781	70.3 (19.1)	6,734	68.3 (15.8)	11,515	69.0 (17.1)
**Lung, trachea (C33**–**34)**	11,258	70.2 (14.1)	12,625	70.3 (13.6)	23,883	70.3 (13.8)
**Skin (C43**–**44)**	16,080	71.0 (26.5)	16,272	71.3 (20.0)	32,352	71.2 (23.0)
Melanoma (C43)	8,787	61.7 (25.0)	8,475	65.1 (19.8)	17,262	63.5 (22.5)
Nonmelanoma^b^ (C44)	7,293	80.5 (17.0)	7,797	77.6 (15.4)	15,090	79.0 (16.4)
**Female breast (C50)**	30,845	61.4 (19.0)	-	-	30,845	61.4 (19.0)
**Prostate (C61)**	-	-	44,592	68.9 (11.7)	44,592	68.9 (11.7)
**Less common cancers**	28,212	67.5 (20.3)	26,203	68.8 (16.4)	54,415	68.2 (18.2)
Mouth, pharynx (C00–14)	1,746	67.1 (20.7)	3,052	64.2 (15.5)	4,798	65.1 (17.5)
Oesophagus (C15)	549	72.5 (15.8)	1,658	68.0 (14.0)	2,207	68.9 (14.8)
Stomach (C16)	1,452	73.1 (20.2)	2,369	70.7 (17.3)	3,821	71.3 (18.4)
Liver (C22)	652	71.4 (18.7)	1,135	66.8 (17.6)	1,787	68.2 (18.3)
Gallbladder, bile ducts (C23–24)	714	71.7 (17.4)	580	69.0 (15.3)	1,294	70.3 (16.4)
Pancreas (C25)	2,844	71.9 (16.5)	2,901	69.4 (14.7)	5,745	70.5 (16.0)
Larynx, epiglottis (C32)	162	65.8 (17.0)	795	68.1 (14.4)	957	67.8 (14.7)
Cervix uteri (C53)	3,237	44.9 (22.2)	-	-	3,237	44.9 (22.2)
Corpus uteri and uterus (C54–55)	6,832	67.5 (16.6)	-	-	6,832	67.5 (16.6)
Ovary (C56, C57.0–4, C48.2)	4,462	65.8 (18.5)	-	-	4,462	65.8 (18.5)
Kidney, excluding renal pelvis (C64)	2,052	67.6 (17.9)	4,340	64.5 (16.5)	6,392	65.5 (17.2)
Urinary tract (C65–68)	3,510	73.1 (17.7)	9,373	72.1 (15.4)	12,883	72.4 (16.0)

*ICD-10* international classification of diseases – 10^th^ revision, *No.*, number, *IQR* interquartile range.

^a^Excluding basal cell carcinoma.

### NCD comorbidity at cancer diagnosis

**Fig. 2:**
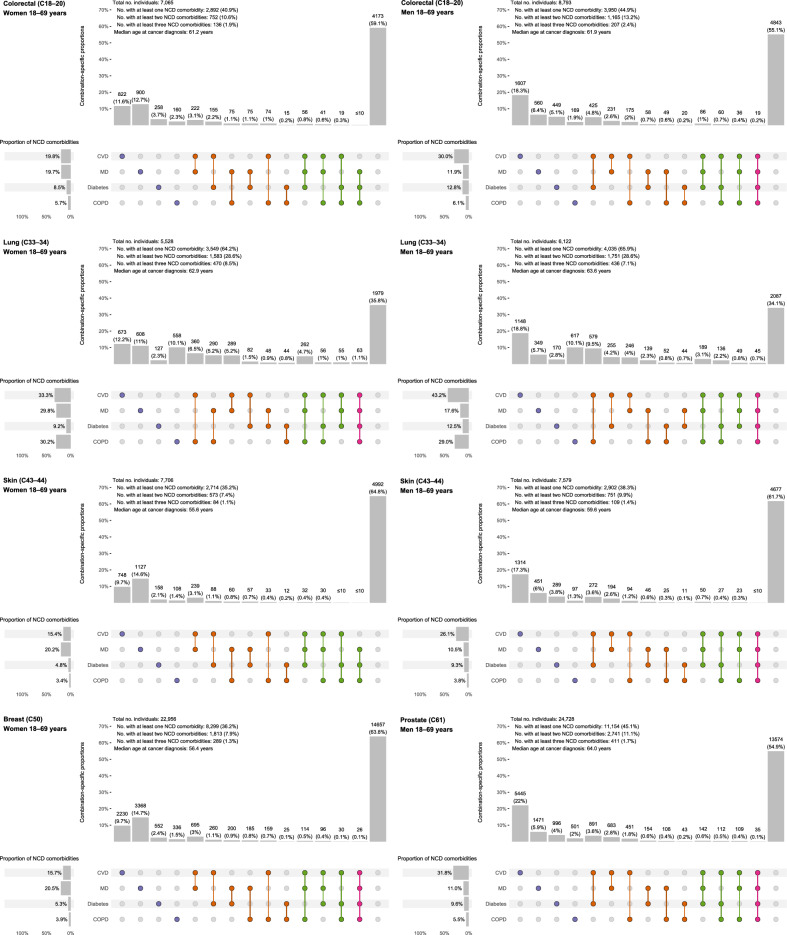
Intersection diagrams showing the prevalence and patterns of noncommunicable disease comorbidities at the time of diagnosis of colorectal, lung, skin, breast and prostate cancers for women and men diagnosed at ages 18–69 years (*n* = 90,477). This shows all observed combinations of noncommunicable disease comorbidities at the time of cancer diagnosis. All single comorbidities are shown, as well as the 10 most common combinations of at least two comorbidities.

**Fig. 3:**
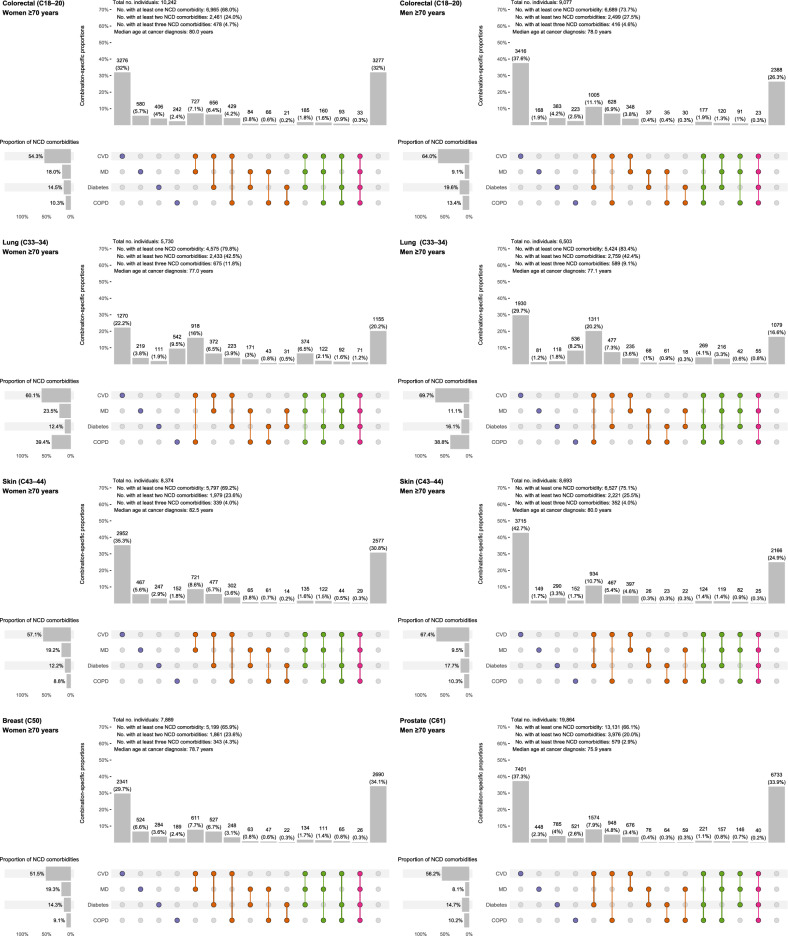
Intersection diagrams showing the prevalence and patterns of noncommunicable disease comorbidities at the time of diagnosis of colorectal, lung, skin, breast and prostate cancers for women and men diagnosed at ages ≥ 70 years (*n* = 76,372). This shows all observed combinations of noncommunicable disease comorbidities at the time of cancer diagnosis. All single comorbidities are shown, as well as the 10 most common combinations of at least two comorbidities.

NCD comorbidity prevalence differed by cancer site, age, and sex. At cancer diagnosis, the prevalence of having at least one NCD comorbidity ranged from 35.2% (women with skin cancer 18–69 years) to 83.4% (men with lung cancer ≥ 70 years). For having at least two NCD comorbidities, the prevalence ranged from 7.4% (women with skin cancer 18–69 years) to 42.5% (women with lung cancer ≥ 70 years), and for having at least three, it ranged from 1.1% (women with skin cancer 18–69 years) to 11.8% (women with lung cancer ≥ 70 years) (Figs. 2 and Fig. 3). Men had higher prevalence of NCD comorbidities (at least one) than women (range women: 35.2%–79.8%, men: 38.3%–83.4%). The most prevalent NCD comorbidity for all strata was CVD (19.8%–69.7%), except for women 18–69 years with skin and breast cancer, where MD was most prevalent (skin: 20.2%, breast: 20.5%). Lung cancer patients had highest prevalence of COPD (29.0%–39.4%). Women had highest prevalence of MD (women: 18.0%–29.8%, men: 8.1%–17.6%) and lowest prevalence of diabetes (women: 4.8%–14.5%, men: 9.3%–19.6%). Prevalence of NCD comorbidity was higher among patients from the older age group (18–69 years: 35.2–66.0%, ≥ 70 years: 65.9–83.4%).

For women, the most prevalent combinations of two NCD comorbidities were CVD with MD for colorectal, skin and breast cancer patients (3.0%–8.6%), and CVD with COPD for lung cancer patients (6.5%–16.0%). For men, the most prevalent combinations were CVD with diabetes for colorectal, skin and prostate cancer patients (3.6%–11.1%), and CVD with COPD for lung cancer patients (9.5%–20.2%). The proportion of patients with three or more NCD comorbidities was low, and lung cancer patients had highest prevalences for the combination of CVD, MD and COPD (3.1%–6.5%). Patterns were similar for the two age groups.

Prevalence and patterns of NCDs were similar for colon and rectal cancer, as well as for melanoma and nonmelanoma skin cancers (Supplementary Figures S1 and S2).

### NCD comorbidity five years post cancer diagnosis

At five years post cancer diagnosis, the probability of being alive with no NCD comorbidities for the most common cancers ranged from 0.7% (men with lung cancer ≥ 70 years) to 46.7% (women with skin cancer 18–69 years), and the probability of being alive with at least one NCD comorbidity ranged from 11.5% to 58.0% (Supplementary File and Supplementary Figures S3–S6). For these cancers, the probability of death at five years ranged from 6.0% to 87.8% and was highest for lung cancer patients, and among patients from the older age group and in men.

In the following, we report the probabilities of having NCD comorbidities five years post cancer diagnosis, conditioning on being alive, including NCDs registered before and after the first cancer diagnosis. Confidence intervals were generally narrow and were omitted for simplicity. We summarize the results for colorectal, lung, skin, female breast and prostate in separate paragraphs with a consistent structure. A lookup table (Supplementary File) and online application [https://ncdapp.onrender.com/] provide results including confidence intervals for all 19 sites, time points and strata.

#### Colorectal cancer

The probability of having at least one NCD comorbidity in colorectal cancer patients ranged from 56.7% (women aged 18–69 years) to 85.3% (men aged ≥ 70 years) (Table 2 and Supplementary Figure S7). The most likely NCD comorbidity was CVD for all strata (range, 31.8%–75.2%), followed by MD for women (23.9%–25.3%), and diabetes for men (15.7%–21.5%). The probability of a second cancer was higher in men than women (women: 6.9%–11.3%, men: 9.3%–16.2%). In general, colon cancer patients had higher NCD comorbidity probability than rectal cancer patients (Supplementary Figures S8 and S9).

**Table 2:** Summary of selected estimated probabilities for noncommunicable diseases for the most common cancers conditioning on being alive five years post cancer diagnosis^a^.

	Cancer type (ICD-10 codes)				
	Colorectal (C18–20)	Lung, trachea (C33–34)	Skin (C43–44)	Female breast (C50)	Prostate (C61)
**Probabilities (%) of no, one, two, or three or more NCD comorbidities**					
**No comorbidities**					
Women diagnosed at ages 18–69 years	43.3%	18.3%	49.7%	45.3%	-
Women diagnosed at ages ≥ 70 years	19.2%	8.3%	19.8%	21.9%	-
Men diagnosed at ages 18–69 years	38.3%	16.8%	44.6%	-	37.3%
Men diagnosed at ages ≥ 70 years	14.7%	5.5%	14.3%	-	20.4%
**One comorbidity**					
Women diagnosed at ages 18–69 years	35.9%	30.4%	35.1%	36.5%	-
Women diagnosed at ages ≥ 70 years	43.7%	25.6%	45.1%	42.0%	-
Men diagnosed at ages 18–69 years	37.1%	28.2%	36.0%	-	41.4%
Men diagnosed at ages ≥ 70 years	42.1%	25.5%	46.5%	-	46.6%
**Two comorbidities**					
Women diagnosed at ages 18–69 years	15.6%	30.8%	11.9%	14.3%	-
Women diagnosed at ages ≥ 70 years	26.6%	39.8%	26.7%	26.5%	-
Men diagnosed at ages 18–69 years	18.2%	34.1%	14.8%	-	16.6%
Men diagnosed at ages ≥ 70 years	31.4%	43.0%	29.2%	-	25.5%
**Three or more comorbidities**					
Women diagnosed at ages 18–69 years	5.2%	20.4%	3.3%	3.8%	-
Women diagnosed at ages ≥ 70 years	10.5%	26.2%	8.4%	9.6%	-
Men diagnosed at ages 18–69 years	6.4%	20.9%	4.6%	-	4.6%
Men diagnosed at ages ≥ 70 years	11.9%	26.0%	10.0%	-	7.5%
**Most likely NCD comorbidity (%)**					
Women diagnosed at ages 18–69 years	CVD (31.8%)	CVD (49.3%)	MD (25.2%)	CVD (30.1%)	-
Women diagnosed at ages ≥ 70 years	CVD (66.2%)	CVD (74.1%)	CVD (67.4%)	CVD (63.8%)	-
Men diagnosed at ages 18–69 years	CVD (43.9%)	CVD (59.6%)	CVD (38.4%)	-	CVD (47.4%)
Men diagnosed at ages ≥ 70 years	CVD (75.2%)	CVD (82.1%)	CVD (76.9%)	-	CVD (70.2%)
**Second most likely NCD comorbidity (%)**					
Women diagnosed at ages 18–69 years	MD (25.3%)	COPD (48.5%)	CVD (25.0%)	MD (26.9%)	-
Women diagnosed at ages ≥ 70 years	MD (23.9%)	COPD (55.5%)	MD (23.1%)	MD (23.5%)	-
Men diagnosed at ages 18–69 years	Diabetes (15.7%)	COPD (52.9%)	MD (13.6%)	-	MD (14.3%)
Men diagnosed at ages ≥ 70 years	Diabetes (21.5%)	COPD (59.8%)	Diabetes (19.4%)	-	Diabetes (16.7%)
**Probability (%) of second cancer**					
Women diagnosed at ages 18–69 years	6.9%	12.9%	7.1%	5.8%	-
Women diagnosed at ages ≥ 70 years	11.3%	15.5%	10.4%	9.9%	-
Men diagnosed at ages 18–69 years	9.3%	14.8%	9.5%	-	5.1%
Men diagnosed at ages ≥ 70 years	16.2%	20.3%	15.7%	-	8.9%

*ICD-10* international classification of diseases – 10^th^ revision, *NCD* noncommunicable disease, *CVD* cardiovascular disease, *MD* mental health disorder, *COPD* chronic obstructive pulmonary disease.

^a^Probabilities are state occupation probabilities estimated at five years using the non-parametric Aalen-Johansen estimator in a multi-state model. Additional details including confidence intervals and results for other cancers and time points can be found in our online application [https://ncdapp.onrender.com/] and in Supplementary File. Probabilities were reported as % in the table.

#### Lung cancer

The probability of having at least one NCD comorbidity in lung cancer patients ranged from 81.7% (women aged 18–69 years) to 94.5% (men aged ≥ 70 years) (Table 2 and Supplementary Figure S10). The most likely NCD comorbidity for all strata was CVD (49.3–82.1%), followed by COPD (48.5–59.8%). The probability of a second cancer was higher in men than women (women: 12.9–15.5%, men: 14.8–20.3%).

#### Skin cancer

The probability of having at least one NCD comorbidity in skin cancer patients ranged from 50.3% (women aged 18–69 years) to 85.7% (men aged ≥ 70 years) (Table 2 and Supplementary Figure S11). For women diagnosed at ages 18–69 years, the most likely NCD comorbidities were MD (25.2%) and CVD (25.0%). For women diagnosed at ages ≥ 70 years, the most likely NCD comorbidity was CVD (67.4%), followed by MD (23.1%). For men, the most likely NCD comorbidity was CVD (18–69 years: 38.4%, ≥ 70 years: 76.9%), followed by MD for men diagnosed at ages 18–69 years (13.6%), and diabetes for men diagnosed at ages ≥ 70 years (19.4%). The probability of a second cancer was higher in men than women (women: 7.1%–10.4%, men: 9.5%–15.7%). Melanoma skin cancer patients had lower NCD comorbidity probability than nonmelanoma patients, except for the probability of second cancer, which was higher in melanoma (Supplementary Figures S12 and S13).

#### Female breast cancer

The probability of having at least one NCD comorbidity in female breast cancer patients ranged from 54.7% (women aged 18–69 years) to 78.1% (women aged ≥ 70 years) (Table 2 and Supplementary Figure S14). The most likely NCD comorbidity was CVD (18–69 years: 30.1%, ≥ 70 years: 63.8%), followed by MD (18–69 years: 26.9%, ≥ 70 years: 23.5%). The probability of a second cancer ranged from 5.8 to 9.9%.

#### Prostate cancer

The probability of having at least one NCD comorbidity in prostate cancer patients ranged from 62.7% (men aged 18–69 years) to 79.6% (men aged ≥ 70 years) (Table 2 and Supplementary Figure S14). The most likely NCD comorbidity was CVD (18–69 years: 47.4%, ≥ 70 years: 70.2%), followed by MD for men diagnosed at ages 18–69 years (14.3%) and diabetes for men diagnosed at ages ≥ 70 years (16.7%). The probability of a second cancer ranged from 5.1 to 8.9%.

### NCD comorbidity 10 years post cancer diagnosis

No changes in NCD comorbidity patterns were observed from five to ten years post cancer diagnosis for all cancers (Supplementary File & online application).

### Other cancers

Results for the less common cancers are presented in the Supplementary File and the online application. Notably, pancreatic cancer patients had the highest five-year post cancer diagnosis mortality among these cancers (range, 83.9%–94.9%). Conditioning on surviving five years, patients with liver cancer, pancreatic cancer, corpus uteri/uterus cancer or kidney cancer had a substantially higher probability of diabetes compared to all other cancer patients (liver: 27.8%–44.8%, pancreas: 26.6%–52.0%, corpus uteri/uterus: 19.9%–21.4%, kidney: 18.7%–23.6%). Finally, cervical cancer patients diagnosed at ages 18–69 years had the lowest probabilities of second cancer (2.7%), CVD (17.0%), diabetes (5.4%), and COPD (3.4%), relative to all other sites and strata, and had the lowest median age at cancer diagnosis (44.9 years).

### Wider sample

In the wider sample analysis, which included individuals with any prevalent cancers diagnosed on a different site (*n* = 246,305), the probability of death increased for nearly all cancer sites, compared to the results above. Conditioning on surviving five years, results remained unchanged for all NCD comorbidities, except second cancer. For the most common cancers, the probability of a prevalent cancer diagnosis on a different site at start of follow-up ranged from 5.7 to 28.1%, and conditioning on surviving five years, the probability of having had at least one other cancer (prevalent or incident) ranged from 10.8 to 45.5% (Supplementary File & online application).

None of the sensitivity analyses described in Supplementary Methods B yielded meaningful differences in the results or interpretation.

## Discussion

In this comprehensive study of more than 200,000 cancer patients, we used data from nationwide-mandatory health registries to identify cancer and NCD comorbidities. At first cancer diagnosis, over one-third of patients had at least one NCD comorbidity, with highest prevalence among men and older patients. The most prevalent NCD comorbidity was CVD, except among younger women with breast and skin cancer for whom it was MD. The most prevalent combinations of two NCD comorbidities at cancer diagnosis were CVD with MD, and CVD with COPD. Five years post cancer diagnosis, the probability of being alive with no NCD comorbidities ranged from 0.7 to 46.7%. Equivalently, 53.3% to 99.3% had either died or were living with at least one NCD comorbidity. Conditioning on surviving five years, the probability of having at least one NCD comorbidity exceeded 50% for all cancer sites and was highest among men and older patients. CVD remained the most likely NCD comorbidity, except among younger women with skin cancer for whom it was MD.

A recent meta-analysis reported comorbidity prevalences ranging from 22.4 to 46.6%^18^, lower than our findings. This can partly be explained by MD not being included as a comorbidity, or by previous studies generally relying on a single data source, often secondary healthcare data, likely underestimating comorbidity prevalence^39^. One study on both common and rarer cancers used primary healthcare data and included a much broader range of comorbidities, finding higher prevalence estimates compared to our study^20^. By combining data from primary and secondary healthcare from national mandatory health registries, we ensured comprehensive comorbidity identification. In Norway, two studies reported the prevalence of NCD comorbidities in cancer patients ranging from 7.1 to 44.1%^40,41^, but comorbidity-specific estimates were not provided. To our knowledge, the present study is the first to provide a comprehensive mapping of NCD comorbidities at cancer diagnosis across 19 different cancer sites.

A recent study has investigated post cancer comorbidity prevalences using primary and secondary healthcare data^26^, covering a wide range of cancer sites and chronic conditions. For the most common cancers, their prevalence estimates were generally consistent with our findings, although they reported higher prevalences of MD. That study relied on cross-sectional data, preventing it from following individual patients over time and from giving time-specific estimates as we did in the present study. To date, no other study used multi-state models to account for the dynamic transitions between different NCDs from cancer diagnosis up to 10 years post cancer diagnosis. Finally, our online application facilitates access to these results.

This study has some limitations. First, data on non-cancer NCD comorbidities were obtained from multiple nationwide mandatory health registries, each covering different levels of healthcare services and disease severity^31^. These variations in NCD definitions and reporting practices across registries may have influenced prevalence estimates, despite efforts to mitigate these differences by combining the data sources. Second, the multi-state models treat comorbidities as progressive states and do not allow transitions back to a comorbidity-free state. Because NCD comorbidities were defined by repeated registry registrations within prespecified windows, our estimates reflect registry-confirmed chronic conditions and will not capture short-lived or single-contact episodes (e.g. brief anxiety or depressive symptoms shortly after diagnosis) that resolve without further registrations. Nonetheless, some chronic conditions may have fewer subsequent registry registrations after diagnosis or treatment initiation, and absence of later registrations should therefore not be interpreted as remission. Third, because historical coverage differed across registries at inception (especially NPR starting in 2008), baseline NCD comorbidity prevalence is likely underestimated in the earliest diagnosis years particularly for CVD and MD (as indicated by our sensitivity analyses) and should be interpreted as conservative. Finally, our estimates capture the registered instances of diagnoses, rather than “prevalences”. Although Norway has universal healthcare coverage, there may be inequalities in healthcare access among different groups or regions of Norway, and some diseases may be detected earlier than others. Nonetheless, given that many of these NCDs are chronic conditions that develop over long periods, pinpointing the exact onset is difficult, regardless of the data sources used.

### Conclusion

Our findings highlight the substantial burden of NCDs among cancer patients, particularly the high prevalence of CVD and mental health disorders. We observed considerable variation in NCD comorbidity patterns by cancer site, age, and sex. An accompanying online application is publicly available to facilitate access to these results, potentially contributing to improved NCD prevention, treatment and surveillance.

## Supplementary Information

Supplementary Figures.

Supplementary File.

Supplementary Methods.

Supplementary Table S1.

## Data Availability

The data that support the findings of this study are available from the Norwegian Health Data Authority and Statistics Norway, but restrictions apply to the availability of these data, which were used under license for the current study. Data are however available from the authors upon reasonable request and with permission of the Norwegian Health Data Authority and Statistics Norway.
