# Supplementary material for: A comprehensive nationwide registry study of noncommunicable disease comorbidities and death in cancer patients in Norway—the NCDNOR project
Source: Sci Rep. 2026 Feb 28;16:11342. doi: 10.1038/s41598-026-41831-6 (PMC13049054; doi:10.1038/s41598-026-41831-6)

Supplementary Figures

A comprehensive nationwide registry study of noncommunicable disease comorbidities and death in cancer patients in Norway—the NCDNOR Project

Simon Lergenmuller PhD; Trude Eid Robsahm PhD; Yngvar Nilssen PhD; Knut Eirik Dalene PhD; Wenche Nystad PhD; Haakon E Meyer MD PhD; Hein Stigum PhD; Vidar Hjellvik PhD; Lars J Kjerpeseth MD PhD; Inger Ariansen MD PhD; Inger Kristin Larsen PhD

Supplementary Figure S1. Intersection diagrams showing the prevalence and patterns of noncommunicable disease comorbidities at the time of diagnosis of **colon, rectal and rectosigmoid, melanoma, and nonmelanoma skin cancers** for women and men diagnosed at ages 18–69 years (*n*=31,143)........ 2

Supplementary Figure S2. Intersection diagrams showing the prevalence and patterns of noncommunicable disease comorbidities at the time of diagnosis of **colon, rectal and rectosigmoid, melanoma, and nonmelanoma skin cancers** for women and men diagnosed at ages ≥70 years (*n*=36,386). 3

Supplementary Figure S3. Probability of being in different states during the five years following **colorectal cancer** diagnosis........ 4

Supplementary Figure S4. Probability of being in different states during the five years following **lung cancer** diagnosis........ 5

Supplementary Figure S5. Probability of being in different states during the five years following **skin cancer** diagnosis......... 6

Supplementary Figure S6. Probability of being in different states during the five years following **female breast and prostate** cancer diagnosis.. 7

Supplementary Figure S7. Probability of being in different comorbidity-specific states during the five years following **colorectal cancer** diagnosis, conditioning on being alive. 8

Supplementary Figure S8. Probability of being in different comorbidity-specific states during the five years following **colon cancer** diagnosis, conditioning on being alive........ 9

Supplementary Figure S9. Probability of being in different comorbidity-specific states during the five years following **rectum, rectosigmoid cancer** diagnosis, conditioning on being alive........ 10

Supplementary Figure S10. Probability of being in different comorbidity-specific states during the five years following **lung cancer** diagnosis, conditioning on being alive. 11

Supplementary Figure S11. Probability of being in different comorbidity-specific states during the five years following **skin cancer** diagnosis, conditioning on being alive........ 12

Supplementary Figure S12. Probability of being in different comorbidity-specific states during the five years following **melanoma skin cancer** diagnosis, conditioning on being alive. 13

Supplementary Figure S13. Probability of being in different comorbidity-specific states during the five years following **nonmelanoma skin cancer** diagnosis, conditioning on being alive........ 14

Supplementary Figure S14. Probability of being in different comorbidity-specific states during the five years following f**emale breast and prostate cancer** diagnosis, conditioning on being alive. 15

Supplementary Figure S1. Intersection diagrams showing the prevalence and patterns of noncommunicable disease comorbidities at the time of diagnosis of colon, rectal and rectosigmoid, melanoma, and nonmelanoma skin cancers for women and men diagnosed at ages 18–69 years (n=31,143). This shows all observed combinations of noncommunicable disease (NCD) comorbidities at the time of cancer diagnosis. All single NCD comorbidities are shown, as well as the 10 most common combinations of at least two NCD comorbidities.

Abbreviations: No., number; NCD, noncommunicable disease; CVD, cardiovascular disease; MD, mental health disorder; COPD, chronic obstructive pulmonary disease.

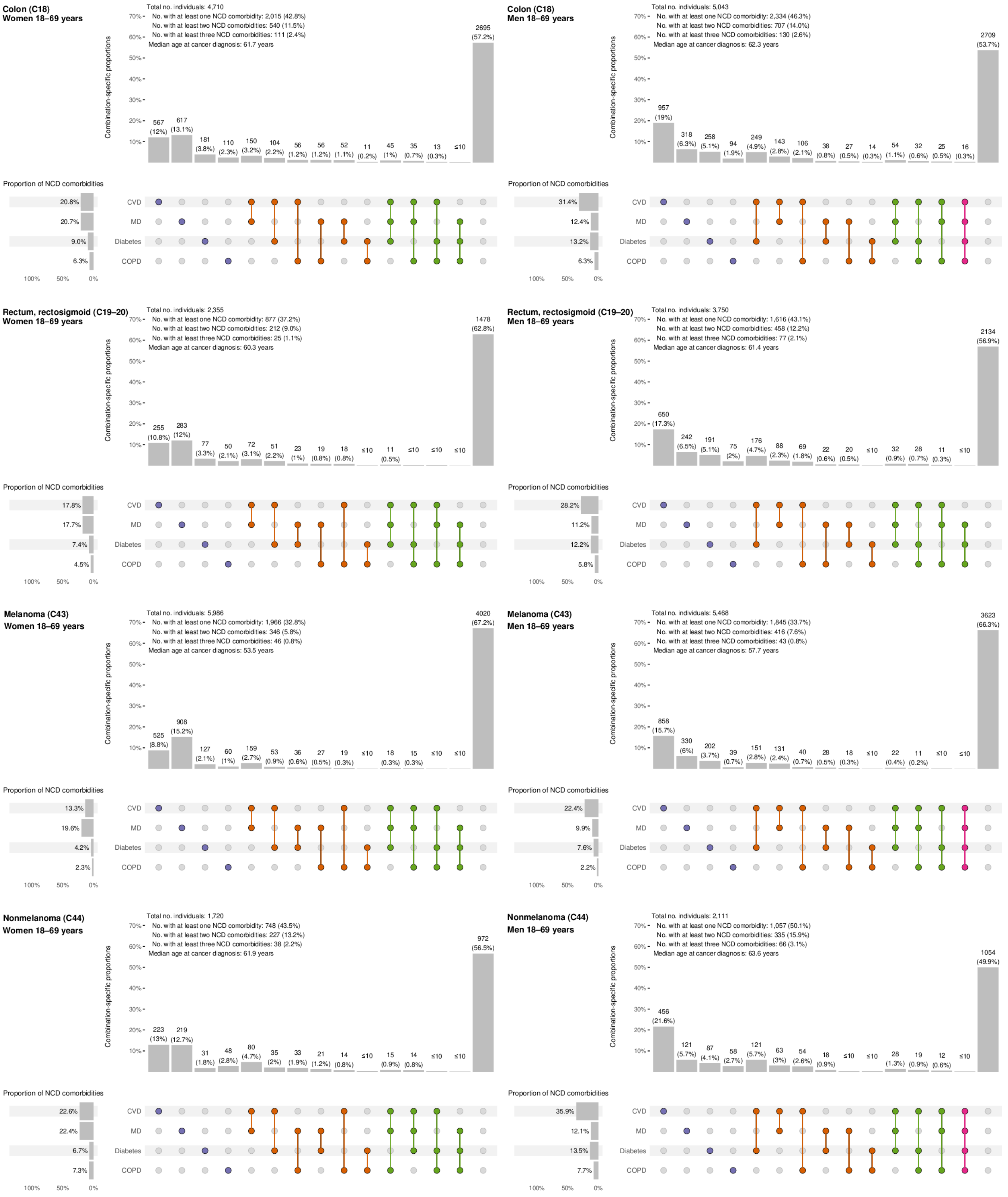

Supplementary Figure S2. Intersection diagrams showing the prevalence and patterns of noncommunicable disease comorbidities at the time of diagnosis of colon, rectal and rectosigmoid, melanoma, and nonmelanoma skin cancers for women and men diagnosed at ages ≥70 years (n=36,386). This shows all observed combinations of noncommunicable disease (NCD) comorbidities at the time of cancer diagnosis. All single NCD comorbidities are shown, as well as the 10 most common combinations of at least two NCD comorbidities.

Abbreviations: No., number; NCD, noncommunicable disease; CVD, cardiovascular disease; MD, mental health disorder; COPD, chronic obstructive pulmonary disease.

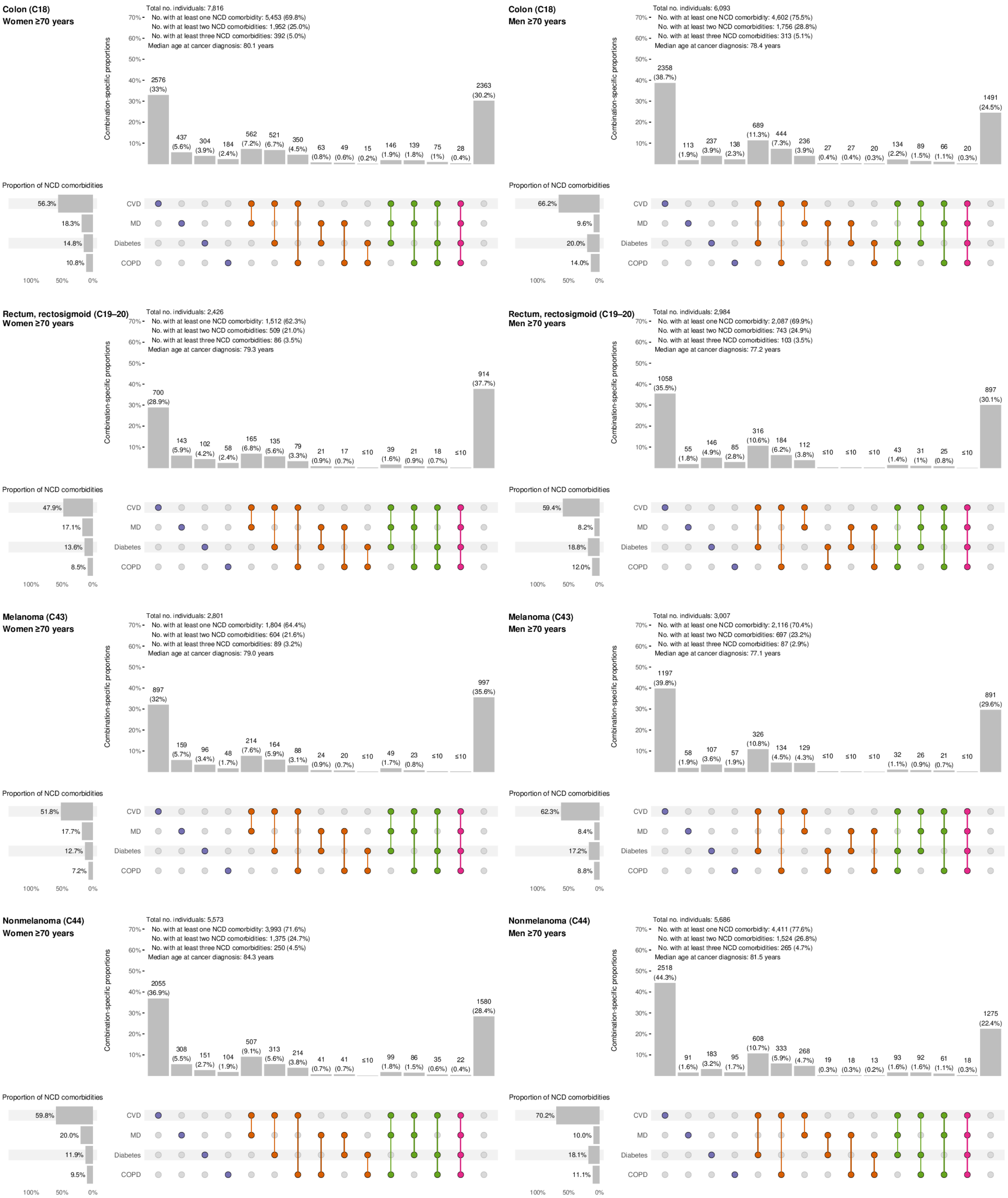

Supplementary Figure S3. Probability of being in different states during the five years following colorectal cancer diagnosis. Four plots are shown – one plot per strata (women, men, diagnosed at ages 18–69 years, diagnosed at ages ≥70 years). The plots give the stacked state occupation probabilities for the following states (from bottom to top): one noncommunicable disease (NCD) comorbidity, two NCD comorbidities, three or more NCD comorbidities, death, and no NCD comorbidities.

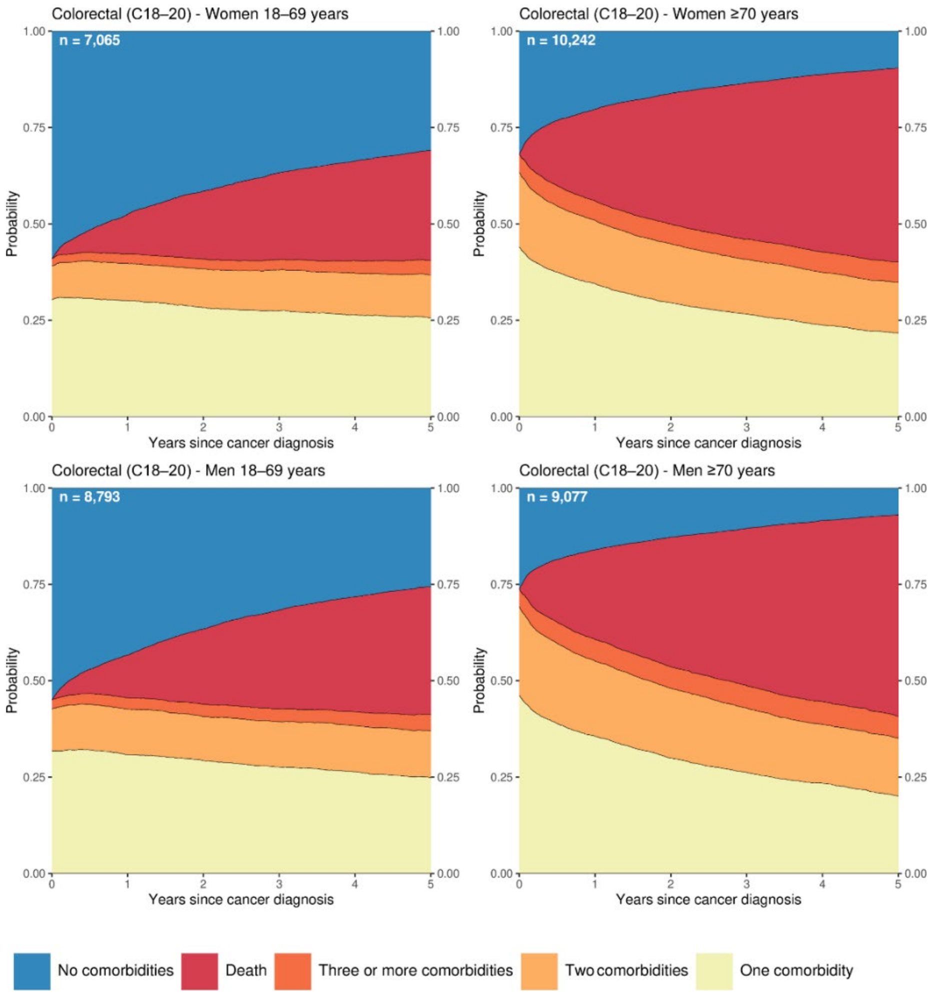

Supplementary Figure S4. Probability of being in different states during the five years following lung cancer diagnosis. Four plots are shown – one plot per strata (women, men, diagnosed at ages 18–69 years, diagnosed at ages ≥70 years). The plots give the stacked state occupation probabilities for the following states (from bottom to top): one noncommunicable disease (NCD) comorbidity, two NCD comorbidities, three or more NCD comorbidities, death, and no NCD comorbidities.

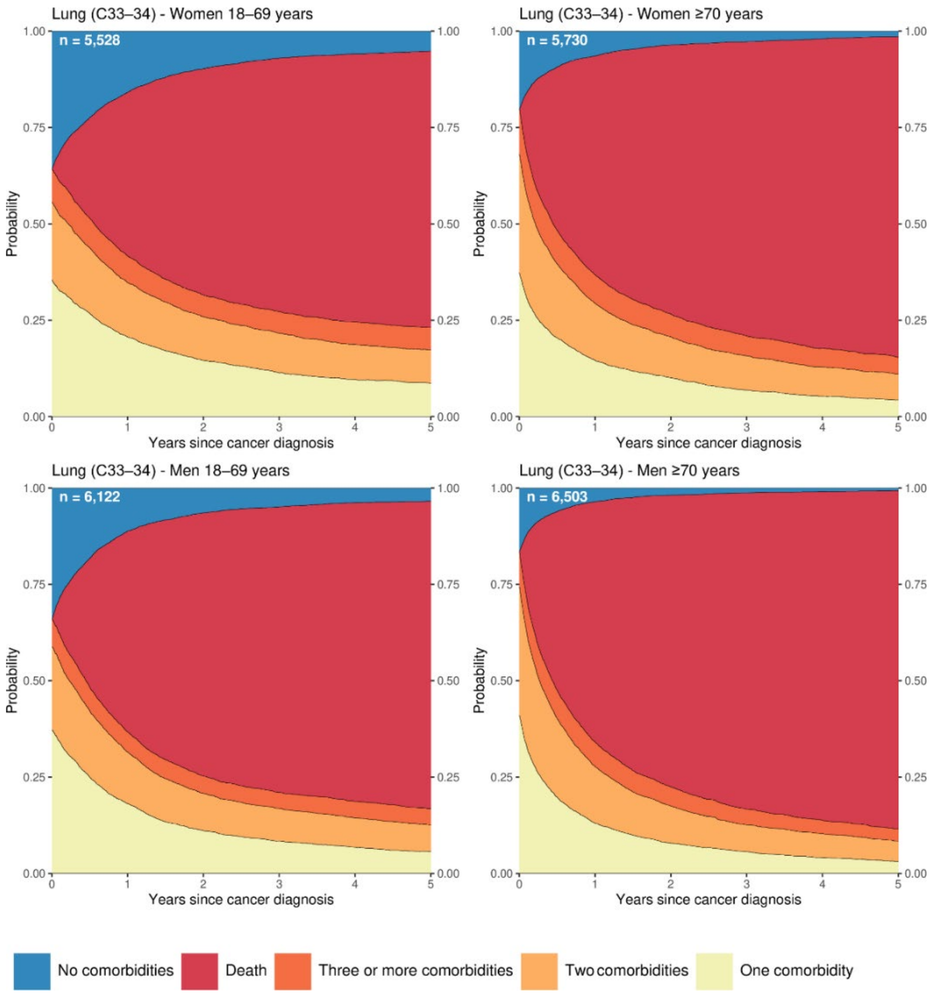

Supplementary Figure S5. Probability of being in different states during the five years following skin cancer diagnosis. Four plots are shown – one plot per strata (women, men, diagnosed at ages 18–69 years, diagnosed at ages ≥70 years). The plots give the stacked state occupation probabilities for the following states (from bottom to top): one noncommunicable disease (NCD) comorbidity, two NCD comorbidities, three or more NCD comorbidities, death, and no NCD comorbidities.

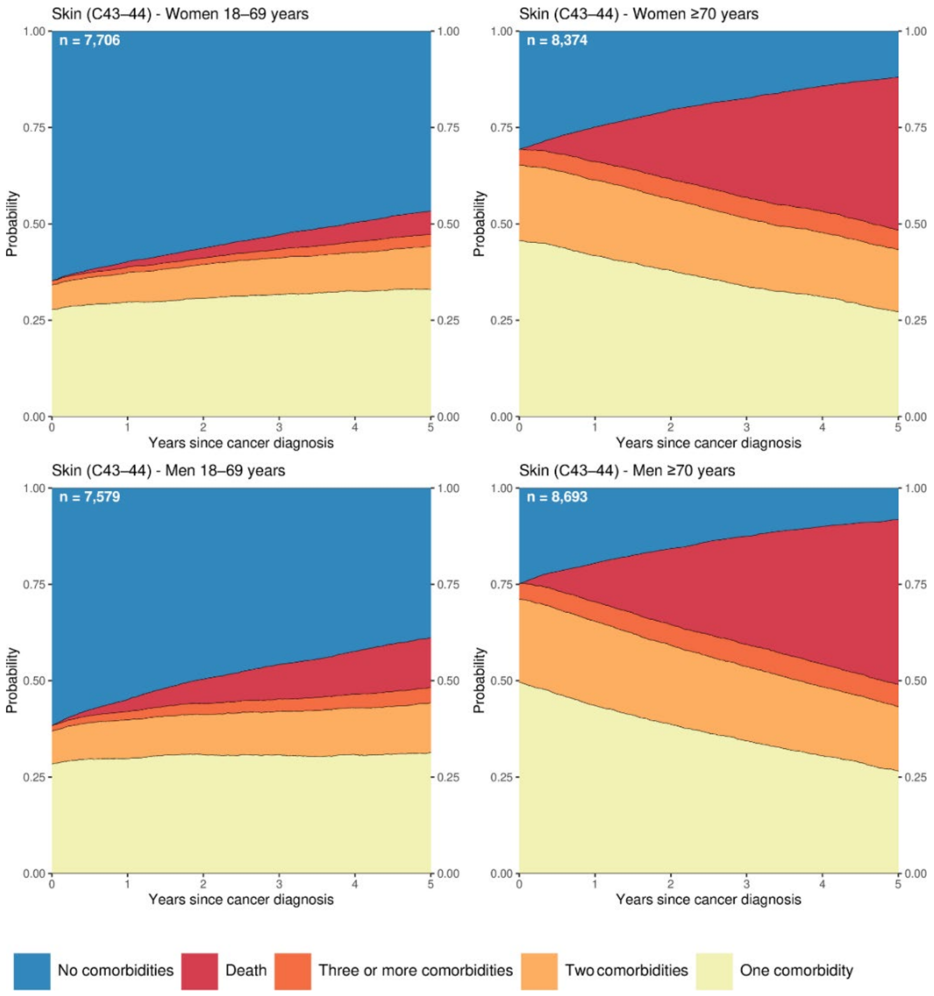

Supplementary Figure S6. Probability of being in different states during the five years following female breast and prostate cancer diagnosis. Four plots are shown – one plot per strata (women [breast cancer], men [prostate cancer], diagnosed at ages 18–69 years, diagnosed at ages ≥70 years). The plots give the stacked state occupation probabilities for the following states (from bottom to top): one noncommunicable disease (NCD) comorbidity, two NCD comorbidities, three or more NCD comorbidities, death, and no NCD comorbidities.

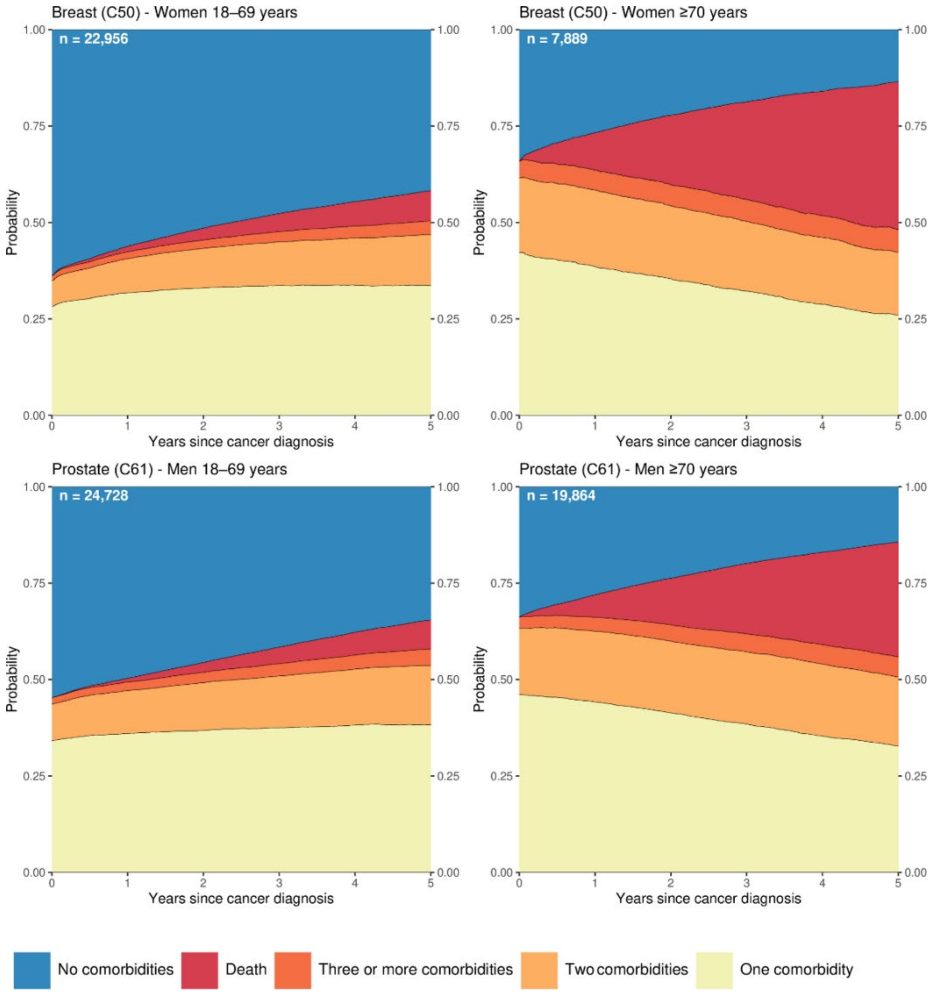

Supplementary Figure S7. Probability of being in different comorbidity-specific states during the five years following colorectal cancer diagnosis, conditioning on being alive. The figure consists of a grid with four plots per row (one per strata: women, men, diagnosed at ages 18–69 years, diagnosed at ages ≥70 years), and five plots per column (one per index-comorbidity: second cancer, cardiovascular diseases, mental health disorders, diabetes, chronic obstructive pulmonary disease). Each plot gives the stacked state occupation probabilities for the following states (from bottom to top): index-comorbidity, non-index comorbidities, no comorbidities.

Abbreviations: CVD, cardiovascular diseases; MD, mental health disorders; COPD, chronic obstructive pulmonary disease.

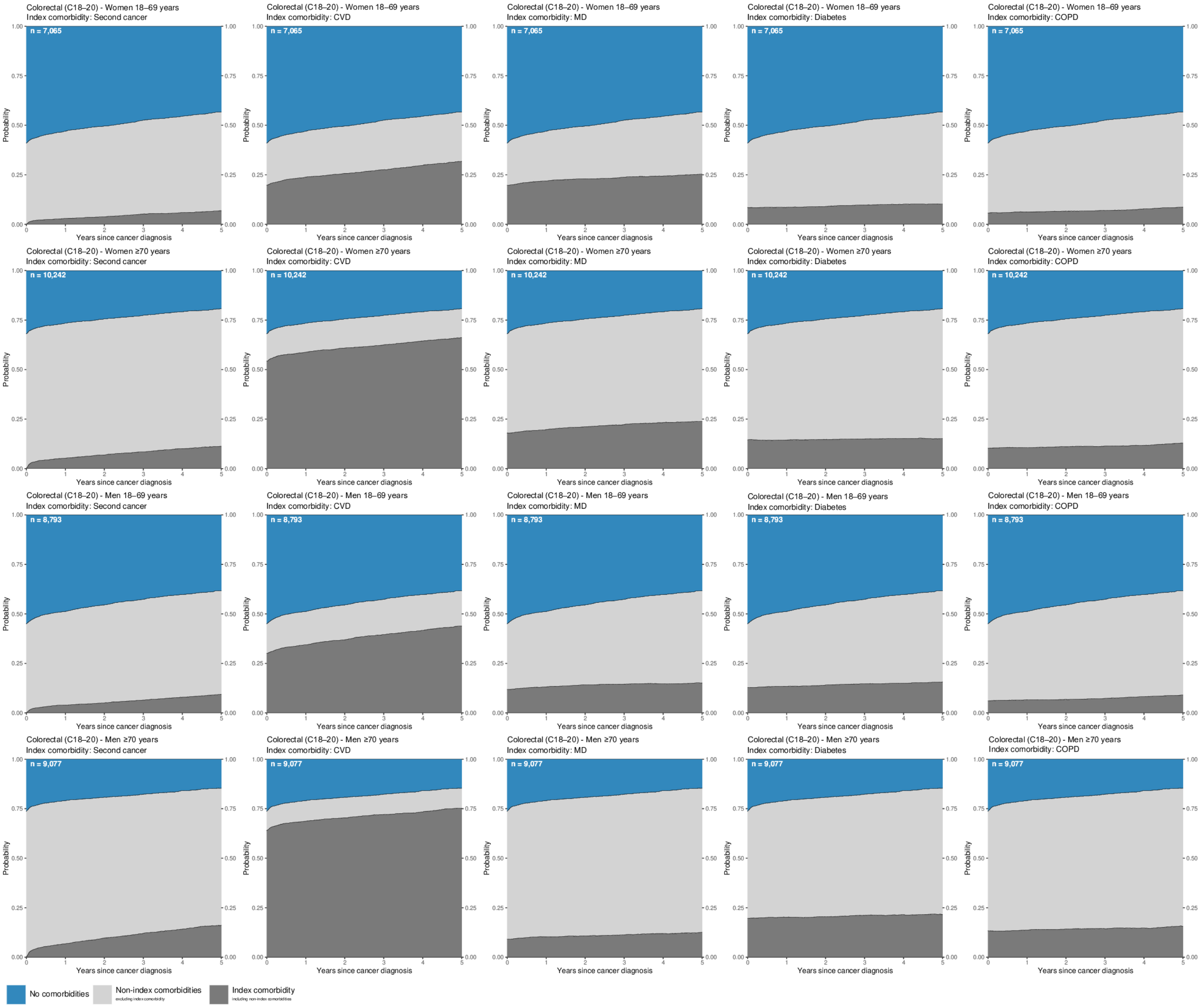

Supplementary Figure S8. Probability of being in different comorbidity-specific states during the five years following colon cancer diagnosis, conditioning on being alive. The figure consists of a grid with four plots per row (one per strata: women, men, diagnosed at ages 18–69 years, diagnosed at ages ≥70 years), and five plots per column (one per index-comorbidity: second cancer, cardiovascular diseases, mental health disorders, diabetes, chronic obstructive pulmonary disease). Each plot gives the stacked state occupation probabilities for the following states (from bottom to top): index-comorbidity, non-index comorbidities, no comorbidities.

Abbreviations: CVD, cardiovascular diseases; MD, mental health disorders; COPD, chronic obstructive pulmonary disease.

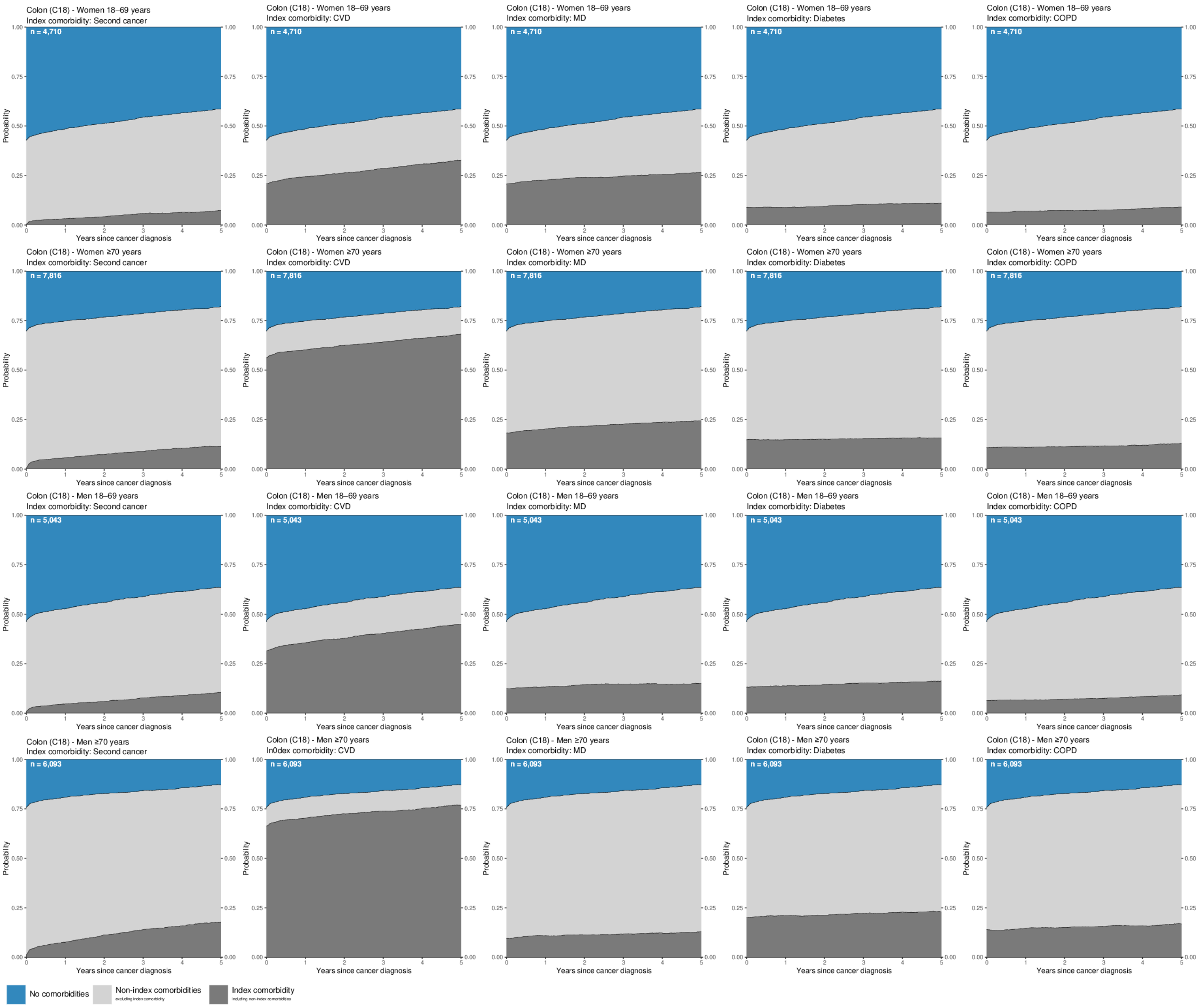

Supplementary Figure S9. Probability of being in different comorbidity-specific states during the five years following rectum, rectosigmoid cancer diagnosis, conditioning on being alive. The figure consists of a grid with four plots per row (one per strata: women, men, diagnosed at ages 18–69 years, diagnosed at ages ≥70 years), and five plots per column (one per index-comorbidity: second cancer, cardiovascular diseases, mental health disorders, diabetes, chronic obstructive pulmonary disease). Each plot gives the stacked state occupation probabilities for the following states (from bottom to top): index-comorbidity, non-index comorbidities, no comorbidities.

Abbreviations: CVD, cardiovascular diseases; MD, mental health disorders; COPD, chronic obstructive pulmonary disease.

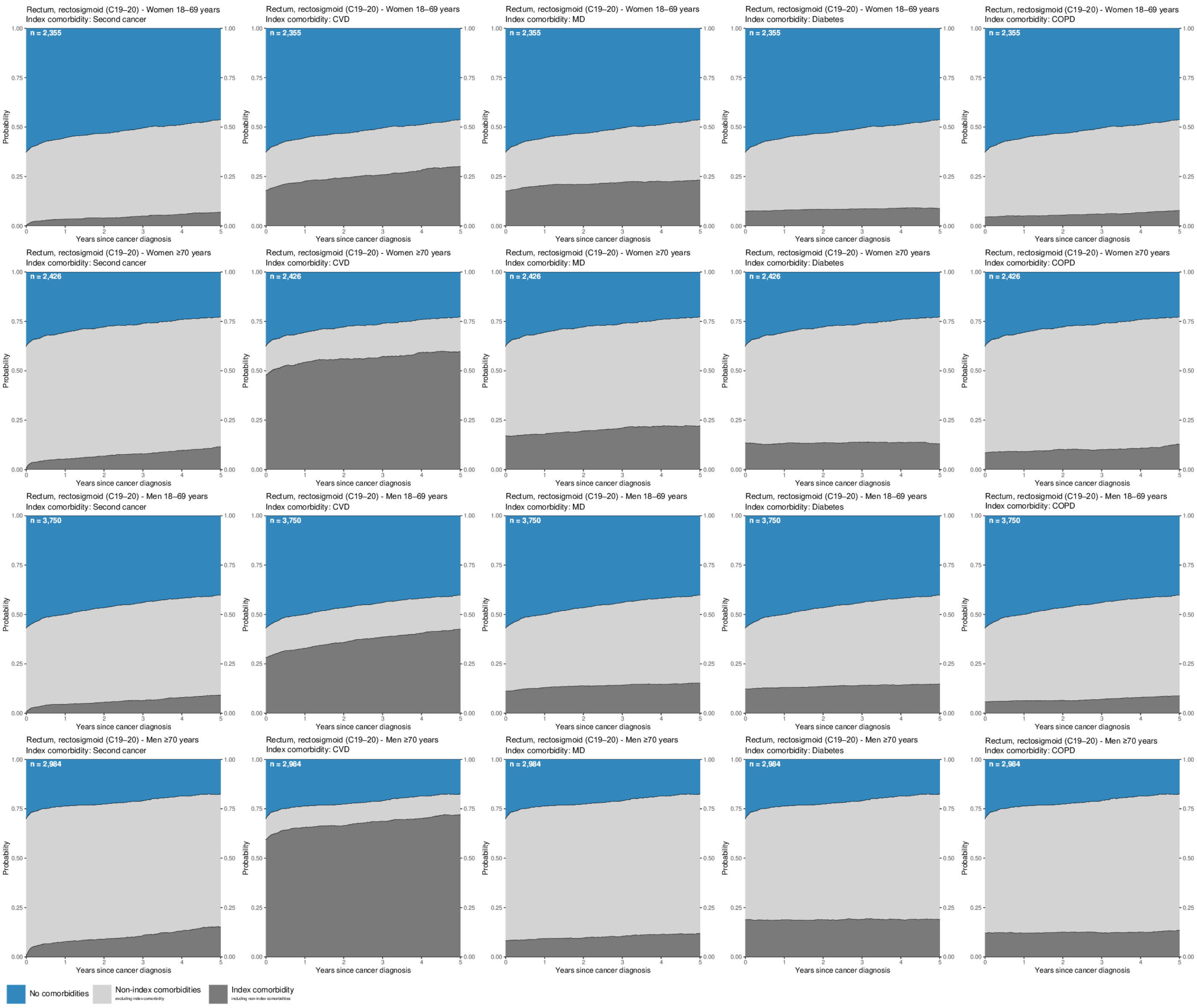

Supplementary Figure S10. Probability of being in different comorbidity-specific states during the five years following lung cancer diagnosis, conditioning on being alive. The figure consists of a grid with four plots per row (one per strata: women, men, diagnosed at ages 18–69 years, diagnosed at ages ≥70 years), and five plots per column (one per index-comorbidity: second cancer, cardiovascular diseases, mental health disorders, diabetes, chronic obstructive pulmonary disease). Each plot gives the stacked state occupation probabilities for the following states (from bottom to top): index-comorbidity, non-index comorbidities, no comorbidities.

Abbreviations: CVD, cardiovascular diseases; MD, mental health disorders; COPD, chronic obstructive pulmonary disease.

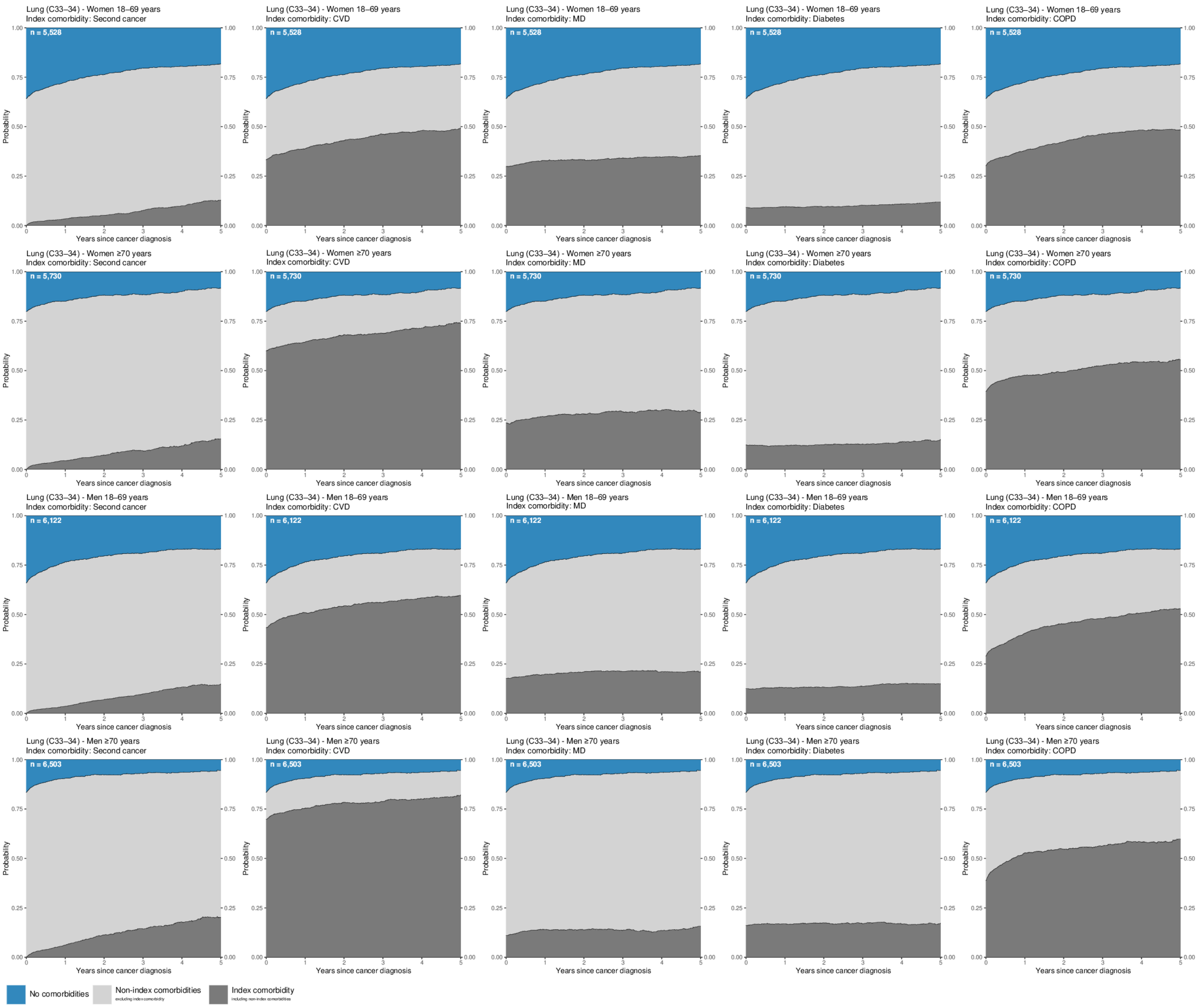

Supplementary Figure S11. Probability of being in different comorbidity-specific states during the five years following skin cancer diagnosis, conditioning on being alive. The figure consists of a grid with four plots per row (one per strata: women, men, diagnosed at ages 18–69 years, diagnosed at ages ≥70 years), and five plots per column (one per index-comorbidity: second cancer, cardiovascular diseases, mental health disorders, diabetes, chronic obstructive pulmonary disease). Each plot gives the stacked state occupation probabilities for the following states (from bottom to top): index-comorbidity, non-index comorbidities, no comorbidities.

Abbreviations: CVD, cardiovascular diseases; MD, mental health disorders; COPD, chronic obstructive pulmonary disease.

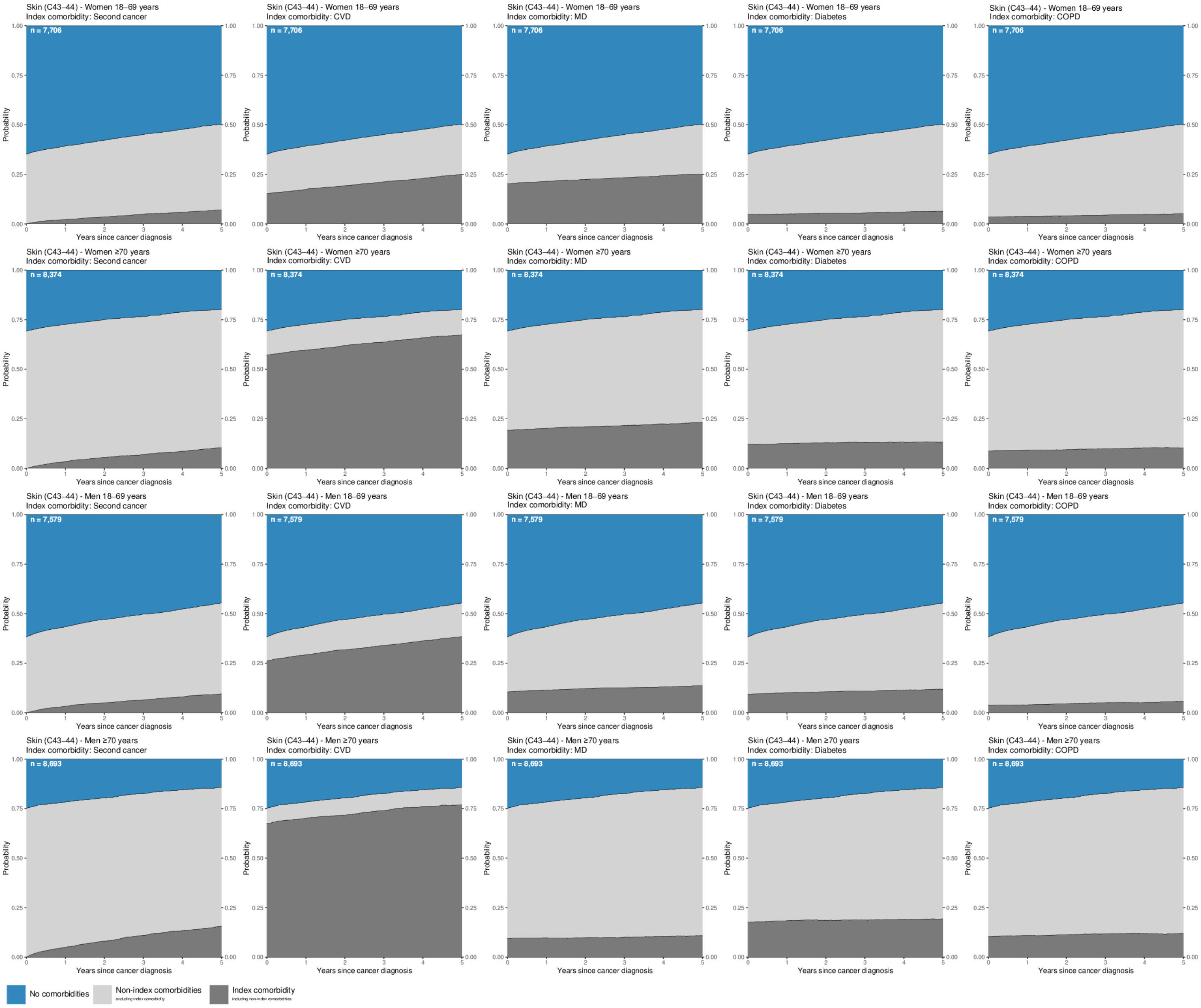

Supplementary Figure S12. Probability of being in different comorbidity-specific states during the five years following melanoma skin cancer diagnosis, conditioning on being alive. The figure consists of a grid with four plots per row (one per strata: women, men, diagnosed at ages 18–69 years, diagnosed at ages ≥70 years), and five plots per column (one per index-comorbidity: second cancer, cardiovascular diseases, mental health disorders, diabetes, chronic obstructive pulmonary disease). Each plot gives the stacked state occupation probabilities for the following states (from bottom to top): index-comorbidity, non-index comorbidities, no comorbidities.

Abbreviations: CVD, cardiovascular diseases; MD, mental health disorders; COPD, chronic obstructive pulmonary disease.

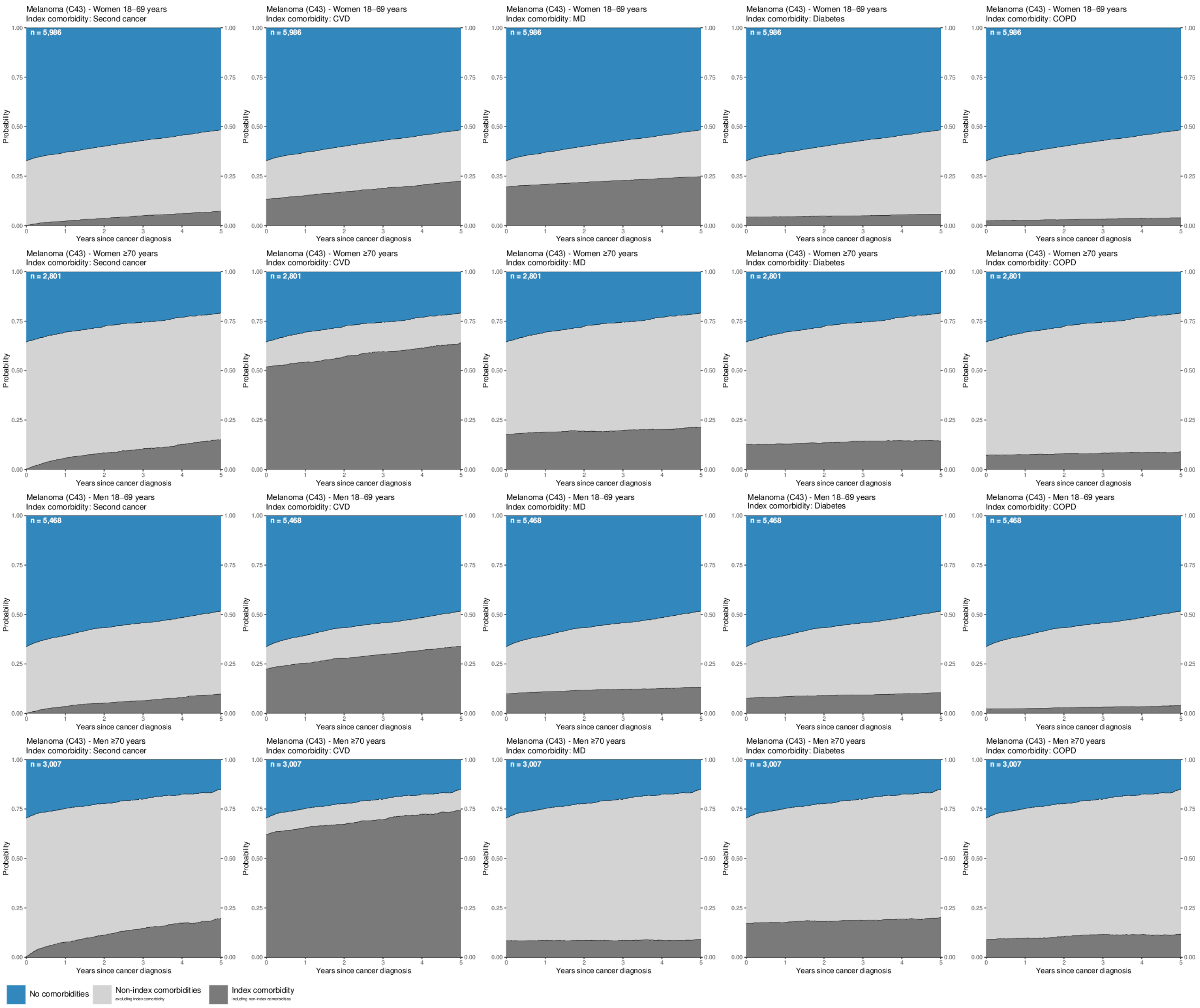

Supplementary Figure S13. Probability of being in different comorbidity-specific states during the five years following nonmelanoma skin cancer diagnosis, conditioning on being alive. The figure consists of a grid with four plots per row (one per strata: women, men, diagnosed at ages 18–69 years, diagnosed at ages ≥70 years), and five plots per column (one per index-comorbidity: second cancer, cardiovascular diseases, mental health disorders, diabetes, chronic obstructive pulmonary disease). Each plot gives the stacked state occupation probabilities for the following states (from bottom to top): index-comorbidity, non-index comorbidities, no comorbidities.

Abbreviations: CVD, cardiovascular diseases; MD, mental health disorders; COPD, chronic obstructive pulmonary disease.

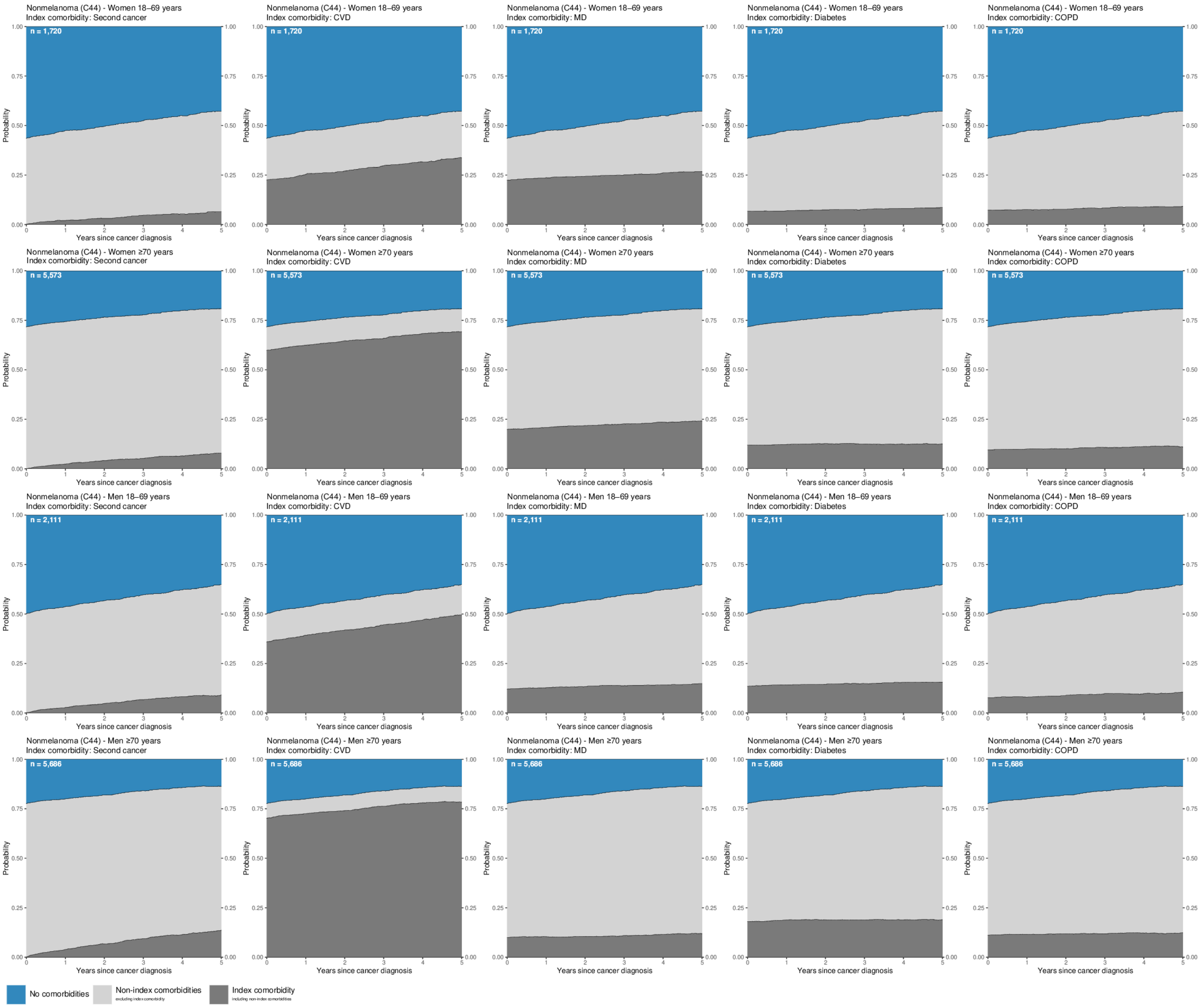

Supplementary Figure S14. Probability of being in different comorbidity-specific states during the five years following female breast and prostate cancer diagnosis, conditioning on being alive. The figure consists of a grid with four plots per row (one per strata: women, men, diagnosed at ages 18–69 years, diagnosed at ages ≥70 years), and five plots per column (one per index-comorbidity: second cancer, cardiovascular diseases, mental health disorders, diabetes, chronic obstructive pulmonary disease). Each plot gives the stacked state occupation probabilities for the following states (from bottom to top): index-comorbidity, non-index comorbidities, no comorbidities.

Abbreviations: CVD, cardiovascular diseases; MD, mental health disorders; COPD, chronic obstructive pulmonary disease.

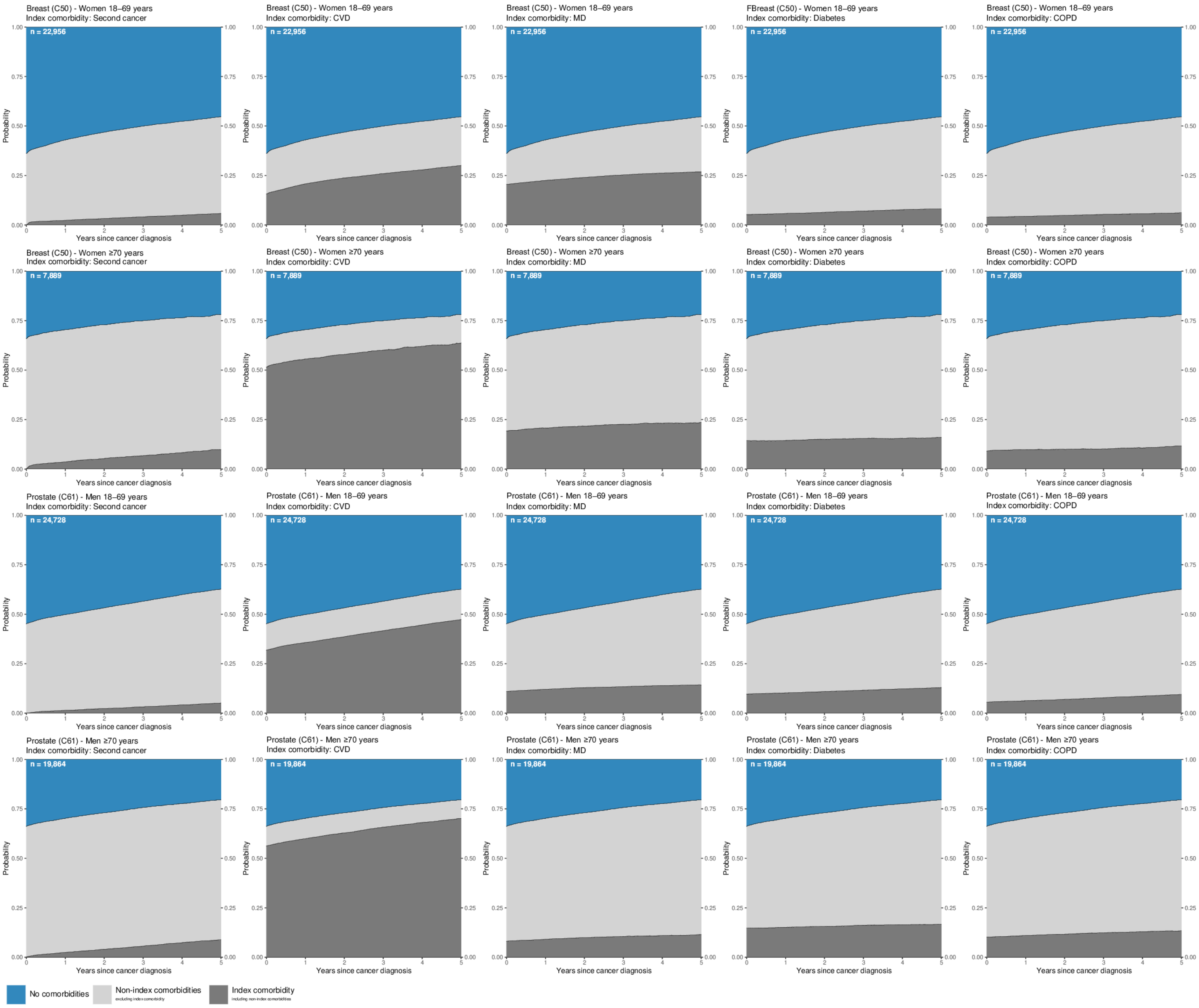

Supplement: Supplementary file 1 — Supplementary Figures. [file 41598_2026_41831_MOESM1_ESM.pdf]
