# Supplementary material for: A comprehensive nationwide registry study of noncommunicable disease comorbidities and death in cancer patients in Norway—the NCDNOR project
Source: Sci Rep. 2026 Feb 28;16:11342. doi: 10.1038/s41598-026-41831-6 (PMC13049054; doi:10.1038/s41598-026-41831-6)
Supplement: Supplementary file 3 — Supplementary Methods. [file 41598_2026_41831_MOESM3_ESM.pdf]

Supplementary Methods

A comprehensive nationwide registry study of noncommunicable disease comorbidities and death in cancer patients in Norway—the NCDNOR Project

Simon Lergenmuller PhD; Trude Eid Robsahm PhD; Yngvar Nilssen PhD; Knut Eirik Dalene PhD; Wenche Nystad PhD; Haakon E Meyer MD PhD; Hein Stigum PhD; Vidar Hjellvik PhD; Lars J Kjerpeseth MD PhD; Inger Ariansen MD PhD; Inger Kristin Larsen PhD

Supplementary Methods A: Details on the multi-state models 2

Supplementary Methods B: Overview of sensitivity analyses 5

Supplementary Methods A: Details on the multi-state models

To estimate the probability of being in any noncommunicable disease (NCD) comorbidity state post cancer diagnosis, we fitted two types of multi-state models (labelled type 1 and type 2), depending on the outcome and states of interest.

In the type 1 multi-state models, the states were: *no NCD comorbidity, second cancer, cardiovascular disease* (CVD), *mental health disorders* (MD), *diabetes*, *chronic obstructive pulmonary disease* (COPD), *two NCD comorbidities*, *three or more NCD comorbidities*, and *death* (Figure A1). For simplicity, the estimated probabilities for *second cancer*, *CVD*, *MD, diabetes* and *COPD* were combined into one state labelled *one NCD comorbidity* (Figure A2). One multi-state model was fitted for each cancer site and strata (men, women, diagnosed at ages 18-69 years, diagnosed at ages ≥70 years).

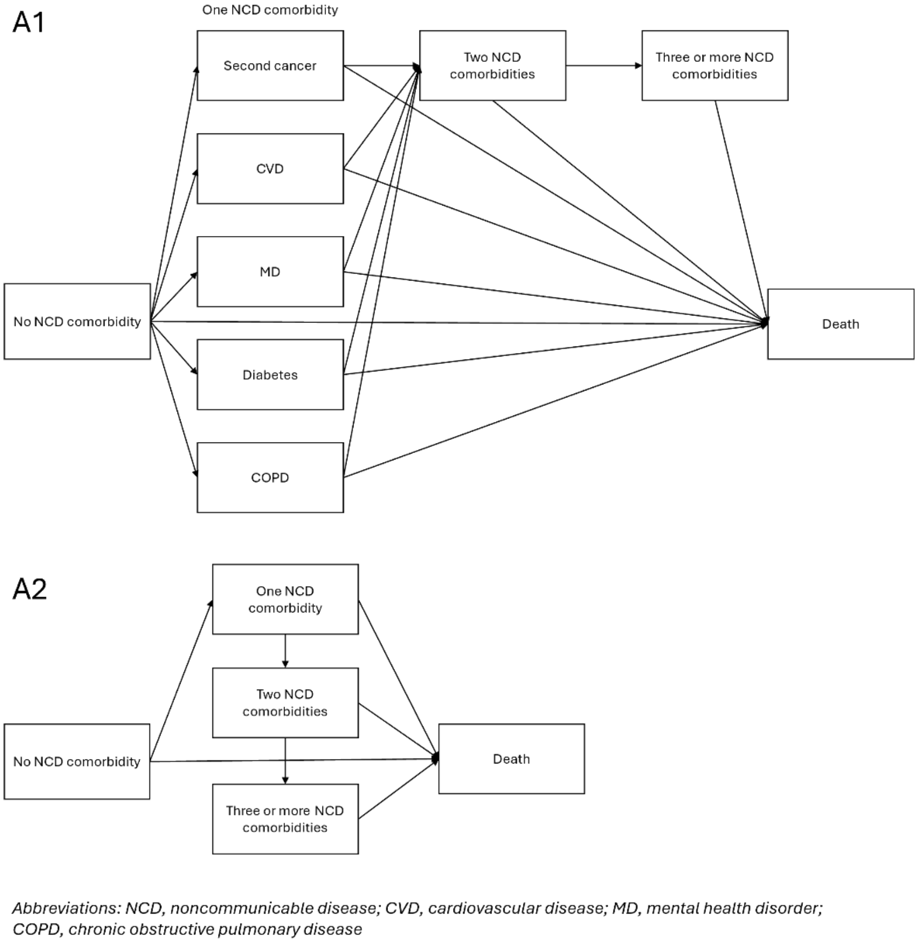

Figure A Complete (A1) and simplified (A2) type 1 multi-state model for NCD comorbidities in cancer patients. The arrows depict the possible transitions between states.

In the type 2 multi-state models, the states were *no NCD comorbidity, index comorbidity* (including non-index comorbidities), *non-index comorbidity* (excluding index comorbidity), and *death* (Figure B1). We use “index” to refer to the specific NCD outcome of interest in the model. For example, when looking at the post cancer diagnosis state probabilities of CVD, the “index” comorbidity is CVD (Figure B2). In this situation, the state probability represents the probability of having CVD *including* other non-CVD comorbidities. An individual in this state may therefore also have other non-CVD (ie

non-index) comorbidities. Similarly, in this example, all individuals in the *non-index comorbidities* state will have at least one of the other NCD comorbidities (excluding CVD). Therefore, one multi-state model was fitted for each index comorbidity of interest (second cancer, CVD, MD, diabetes, and COPD), and for each cancer site and strata (as with type 1).

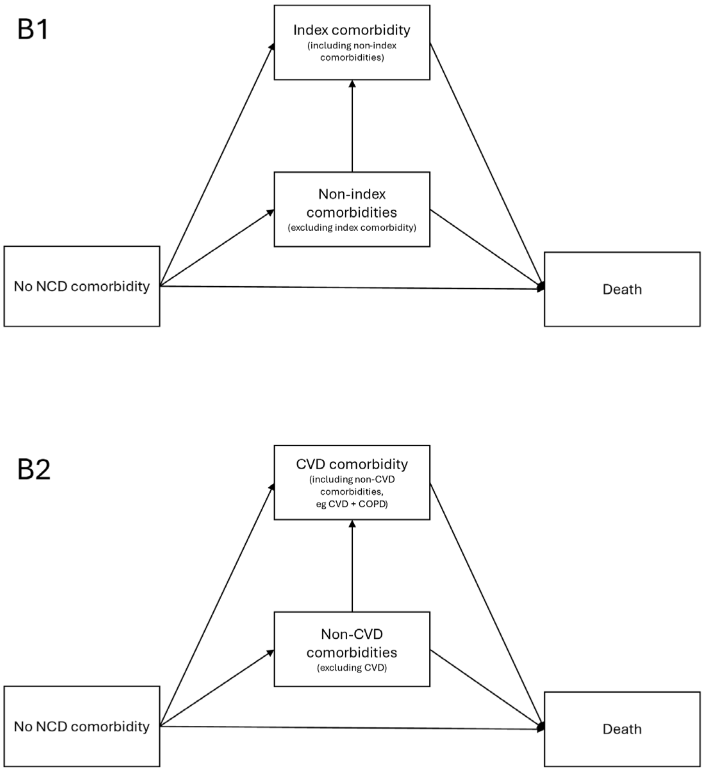

Abbreviations: NCD, noncommunicable disease; CVD, cardiovascular disease; COPD, chronic obstructive pulmonary disease

Figure B Type 2 multi-state model for NCD comorbidities in cancer patients, generic (B1) and exemplified with CVD (B2). The arrows depict the possible transitions between states.

Note that individuals can essentially start in any state, except *death*. In the main analysis, individuals have (by definition) no additional cancer and may therefore not start in the *second cancer* state. In the analysis on the wider sample defined as "*all first primary cancers*" (which also includes all individuals who have previously been diagnosed with a cancer in a different organ), individuals may also start in the *second cancer* state.

Estimation of state probabilities

Let $X(t)$ denote the state of an individual at time t, and $\mathbf{P}(s,t)$ the transition probability matrix with elements $P_{hj}(s,t) = P(X(t) = j | X(s) = h)$ representing the probability of going from state h to state j in the time interval $(s,t]$ (either directly or through other states). Given the transition probability matrix, we can calculate the state probabilities (probability of being in state j at time t) by

$$\hat{P}(X(t) = j) = \sum_k \hat{P}_{kj}(0,t) \cdot \hat{P}(X(0) = k). \quad (1)$$

$P(X(0) = k)$ is estimated by the proportion of subjects entering the study in state k at time zero, and the elements of the transition probability matrix $\hat{P}_{kj}(0, t)$ are estimated with the matrix product formula. Practical details on the estimation can be found elsewhere[1,2]. Briefly, we used a fully non-parametric Aalen-Johansen estimator to estimate state occupation probabilities. We first estimated transition-specific cumulative hazards using the Nelson-Aalen estimator and then obtained transition probability matrices using the probtrans function from the mstate package. State occupation probabilities were then computed using expression (1). We show the estimates $\hat{P}(X(t) = j)$ for t = 0, 1, 2, 3, 4, 5, and 10 years since the first cancer diagnosis.

For the estimation of the state occupation probabilities, we assumed independent censoring. This is a reasonable assumption, as data was almost exclusively censored administratively in 2019 (>99% was independent censoring).

Multi-state models generally rely on Markov assumption, requiring that the instantaneous risk of transition to any other state only depends on the current state and not the state history. However, for state probabilities, the estimator has been proven to be consistent also when the Markov assumption is violated,[3] we therefore did not test the Markov assumption.

We also provide estimates conditioned on being alive. These are simply obtained at each time point t by dividing the probability of being in the specific (alive) state at time t by the probability of being alive (one minus the probability of death) at time t.

Data privacy considerations for results availability

For some cancer sites, some states may include few individuals at specific time points. To protect individuals' privacy, we remove results for states for which we estimate there are too few (typically fewer than 10) or too many (typically close to 100%) individuals. However, it may still be possible to infer an approximation of the number of individuals in these states by using the state occupation probability estimates for other states. To prevent such reverse-estimation, we apply the following rules:

Rule 1: If probability estimates together with the number of individuals in a stratum indicate too few or too many individuals in the *no comorbidity* **and/or** death states, we delete all probability estimates at that time point and all subsequent time points, except the probabilities for *index comorbidity* conditioned on being alive five years post cancer diagnosis.

Rule 2: If probability estimates together with the number of individuals in a stratum indicate too few or too many individuals in the *one comorbidity*, *two comorbidities* **or** *three or more comorbidities* state, we delete the probability estimates for all these three states at that time point and all subsequent time points, including those conditioned on being alive five years post cancer diagnosis.

Rule 3: If probability estimates together with the number of individuals in a stratum indicate too few or too many individuals in the *index comorbidity* state, we delete the probability estimates for that state at that time point and at least one adjacent time point, including results conditioned on being alive. However, if Rule 1 has already resulted in deletion at the same timepoints, we may retain the probability estimates for *index comorbidity* conditioned on being alive five years post cancer diagnosis.

For less common cancers, these rules may result in the deletion of a substantial number of results. However, since the age-specific number of individuals is never shown for these cancer sites, exceptions were made on a case-by-case basis to minimize unnecessary deletions. These exceptions are not detailed here.

Supplementary Methods B: Overview of sensitivity analyses

We conducted sensitivity analyses to evaluate the impact of the various choices made in the study. We briefly describe the analyses here, and additional information may be provided upon reasonable request.

More detailed multi-state models

In the paper, to avoid transitions with very few events and to reduce the computational demand, some states were collapsed into one. For example, all comorbidities registered after the third comorbidity were ignored, and individuals stayed in the *three or more comorbidities* state until end of follow-up, death or emigration. Similarly, in the models estimating the *non-index comorbidities* we assumed that once an individual entered the *non-index comorbidity* state, they will stay in that state until they transition to *index comorbidity* or *death* or reach end of follow-up. In practice, the proportion of individuals for whom these states were ignored was small, and analyses in which we allow these states to contribute to the estimation gave nearly identical results.

Additional age strata

We used 70 years as the cut-off for age, as cancer is the most important cause of death in the population <70 years (premature death).[4] Given the wide range of the younger age group (18-69 years), we also conducted sensitivity analyses by further stratifying this group into patients diagnosed at ages 18-49 and 50-69 years, as well 18-59 and 60-69 years. While the overall patterns remained consistent across the strata, the probabilities of comorbidities and death were, as expected, lower in the youngest groups and higher in the older groups. The results were similar when comparing the 18-49 and 18-59 groups, as well as the 50-69 and 60-69 groups.

Registry availability and baseline comorbidity

The inception of the most recent data source (Norwegian Patient Registry, NPR) was 2008, and the follow-up started in 2009 to allow for a more correct classification of the NCDs (as prevalent or incident) registered during 2008. Because cancers diagnosed early in follow-up have less pre-diagnosis history than cancers diagnosed later, baseline comorbidity prevalence may be underestimated in the earlier diagnosis years. This can occur if the first recorded registration falls before the available registry history, and because some chronic conditions may have fewer subsequent registry registrations after diagnosis or treatment initiation. To assess the impact of limited historical coverage on the classification of NCDs as prevalent, we therefore re-estimated the baseline NCD prevalence in later diagnosis years, while restricting historical registry data to mimic the 2009 availability (NPR: 1-year history; Norwegian database for Control and Payment of Health Reimbursement: 3-year history; The Norwegian Prescription Database: 5-year history) and compared this with estimates using longer registry history. Under this restriction, baseline prevalence decreased for most NCDs, with the largest differences observed for CVD and MD (for example, in 2016 across all major cancers: 7.5 percentage points for CVD, 3.4 percentage points for MD, 1.3 percentage points for COPD and 0.9 percentage points for diabetes). These reductions attenuated substantially when using longer history availability, suggesting baseline prevalence estimates in the earlier years are conservative. Additionally, we compared the baseline NCD proportions across diagnosis years (2009-2019) using for each year the full available history. These distributions remained stable throughout, suggesting that the overall patterns by cancer site, age, and sex are not driven by the increasing amount of historical data.

Impact of assigning NCD registration date to the first registration

We adopted a two-registration rule for non-cancer NCDs to better capture chronic conditions and to avoid classifying single, short-lived episodes as NCD comorbidities. The second registration is used to confirm the condition, and we use the date of the first registration to better approximate onset. As a result, patients who die before a confirming registration may be misclassified as not having the NCD comorbidity, which could underestimate NCD comorbidity occurrence, particularly early after cancer diagnosis in cancers with high mortality. In addition, because confirmation requires a second registration at least 75 days after the first, classification depends on surviving long enough to be confirmed and may therefore attenuate early mortality shortly after the assigned onset date.

To assess the potential impact on timing among confirmed cases, we conducted a sensitivity analysis using the second confirming registration date as the date of NCD comorbidity onset instead of the first registration date. Differences in state occupation probabilities at 5 years were small across cancers and strata (generally <1 percentage point), with the largest difference observed for COPD in lung cancer (maximum 2.2 percentage points). Differences in probability of death five years post diagnosis were ≤0.1 percentage points. For the probabilities of 0, 1, 2, ≥3 NCD comorbidities conditioning on being alive five years post diagnosis, using the confirming registration as the onset date produced only small changes, with differences ranging from -1.5 to +1.1 percentage points (when doing “first registration” minus “second registration”). Differences were largest for lung cancer and followed an expected pattern: slightly lower probabilities of no NCD comorbidity and slightly higher probability of one or more NCD comorbidities.

References

1. Hoff R, Corbett K, Mehlum IS, et al. The impact of completing upper secondary education - a multi-state model for work, education and health in young men. *BMC Public Health* 2018; **18**(1): 556.
2. de Wreede LC, Fiocco M, Putter H. The mstate package for estimation and prediction in non- and semi-parametric multi-state and competing risks models. *Comput Methods Programs Biomed* 2010; **99**(3): 261-74.
3. Datta S, Satten GA. Estimation of integrated transition hazards and stage occupation probabilities for non-Markov systems under dependent censoring. *Biometrics* 2002; **58**(4): 792-802.
4. Sung H, Ferlay J, Siegel RL, et al. Global Cancer Statistics 2020: GLOBOCAN Estimates of Incidence and Mortality Worldwide for 36 Cancers in 185 Countries. *CA Cancer J Clin* 2021; **71**(3): 209-49.
