# Supplementary material for: A comprehensive nationwide registry study of noncommunicable disease comorbidities and death in cancer patients in Norway—the NCDNOR project
Source: Sci Rep. 2026 Feb 28;16:11342. doi: 10.1038/s41598-026-41831-6 (PMC13049054; doi:10.1038/s41598-026-41831-6)
Supplement: Supplementary file 4 — Supplementary Table S1. [file 41598_2026_41831_MOESM4_ESM.pdf]

A comprehensive nationwide registry study of noncommunicable disease comorbidities and death in cancer patients in Norway—the NCDNOR Project

Simon Lergenmuller PhD; Trude Eid Robsahm PhD; Yngvar Nilssen PhD; Knut Eirik Dalene PhD; Wenche Nystad PhD; Haakon E Meyer MD PhD; Hein Stigum PhD; Vidar Hjellvik PhD; Lars J Kjerpeseth MD PhD; Inger Ariansen MD PhD; Inger Kristin Larsen PhD

Supplementary Table S1. Diagnosis- and ATC-codes used to identify individuals with noncommunicable disease comorbidities[a].

NCD	ICD-10 (NPR, CPHR, CRN)	ICPC-2 (CPHR)	ATC (NorPD)	Time window
CVD	I00–I99 (except I00–I02, I10, I269, I281–I309, I312–I339, I40–I41, I76, I781–I789, I80–I81, I822, I824, I826, I828, I829, I83, I86, I871–I872, I878–I880, I888–I889, I95, I96, I973–I999)	K70–K99 (except K86, K88, K93, K95, K96, K99)	B01, C, (except C01C, C05, C10), reimbursed with an ICD-10 or ICPC-2 code corresponding to the CVD definition for NPR and CPHR	75–365 days
Mental health disorders (depression, anxiety)	F32–F34, F40–F42	P74, P76	N06A reimbursed with F32–F34, F40–F42, –F3, –F4 or P74, P76, –73, –74	75–365 days
Diabetes	E10–E14	T89–T90	A10A, A10B (except prescriptions reimbursed for gestational diabetes (ICD10 O244; ICPC2 W85))	75–365 days
COPD	J43–J44	R95	R03AC R03AK, R03AL, R03BB, R03DA, R03DX07 (except R03AC02, R03AC03, R03AC04), reimbursed with J43–44 or R95	30–730 days
Cancer	C00–96, D45–47 (CRN only)	-	-	-

Abbreviations: NCD, Non-communicable diseases; ICD-10, International Classification of Diseases – 10th revision; ICPC-2, International Classification of Primary Care - 2nd edition; ATC, Anatomical Therapeutical Chemical system; NPR, Norwegian Patient Registry; CPHR, Norwegian database for Control and Payment of Health Reimbursement; CRN, the Cancer Registry of Norway; NorPD, The Norwegian Prescription Database; CVD, Cardiovascular disease; COPD, Chronic obstructive pulmonary disease.

* The inception of the most recent data source (NPR) was 2008, but the follow-up started in 2009. The added 'buffer year' allows a more correct classification of the NCDs (as prevalent or incident) registered during this year. The buffer year concerns registry inception and baseline classification, whereas the NCD-specific time windows are part of the case definition requiring confirmatory registrations and are independent of the buffer year. To be classified as a chronic non-cancer NCD comorbidity, at least two neighboring registrations needed to occur within an NCD-specific time window (see table), and the time at the first registration was used. For MD, diabetes and COPD, the neighboring registrations could occur in any of the three registries. For CVD, a registration in NPR did not need a neighboring registration, a registration in CPHR needed a neighboring registration in any of the three registries, and a registration in NorPD needed a neighboring registration in CPHR or NPR. Year of inception of the other data sources: CPHR, 2006; NorPD, 2004; CRN, 1953.
